# Rational Design of Ionomer Microstructures for Thermally Reprocessable Materials with Creep Resistance and Recoverability

**DOI:** 10.1021/jacsau.5c01317

**Published:** 2025-11-29

**Authors:** Chia-Chi Tsai, Hanwen Fan, Yuxiao Zhou, Shuyi Xie

**Affiliations:** † Department of Chemical Engineering, 14736 Texas A&M University, College Station, Texas 77843, United States; ‡ Department of Mechanical Engineering, Texas A&M University, College Station, Texas 77843, United States

**Keywords:** ionomer, block copolymer, creep resistance, recoverability, self-assembly, structure−property relationship, reprocessable elastomer, sustainability

## Abstract

Designing dynamic polymer networks that resist creep while remaining reprocessable is a central challenge in sustainable polymeric materials development. Here, we report charge-neutral diblock copolymers (i.e., ionomers) with 18 mol % ammonium chloride that combine high creep resistance and recoverability (>90% recovery after five creep cycles) with thermal processability (compression moldable at 80 °C), outperforming conventional statistical ionomers that soften at elevated temperatures due to ion dissociation. Unlike the 1–3 nm ionic clusters formed in statistical ionomers, these diblock ionomers self-assemble into an inverse hexagonal (iHEX) morphology where glassy ionic domains form the continuous matrix and rubbery neutral domains form the cylinders. The rigid ionic scaffold and large interdomain spacing (>30 nm) substantially extend chain pull-out times and interdomain diffusion, imparting elasticity, while the unentangled flexible blocks within the rubbery cylinders enable processability. By demonstrating that precise control over ion distribution can convert a thermoplastic-like ionomer into a reprocessable elastomer, this work establishes a general design principle for creating nanostructured dynamic polymers with enhanced mechanical integrity, recoverability, and sustainability.

## Graphical abstract

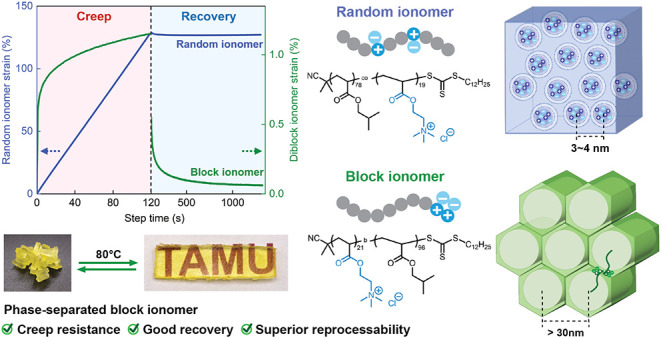

## 1 Introduction

Thermosetting polymers and thermoplastic elastomers (TPEs) represent two dominant classes of polymeric materials used in structural applications. Thermosets form covalently cross-linked networks that exhibit excellent dimensional stability, mechanical strength, and creep resistance, making them ideal for load-bearing and high-temperature applications. However, their permanent network structure renders them nonreprocessable and difficult to recycle.^1^ In contrast, thermoplastic elastomers possess a physically cross-linked and microphase-separated structure, combining elasticity with melt processability. This enables thermal reprocessing, reshaping, and recycling, which are critical for sustainable manufacturing.^2^ However, due to the dynamic and reversible nature of their physical cross-links, TPEs often suffer from long-term creep and poor stress retention under sustained load, limiting their use in high-performance applications where dimensional integrity is critical.

Bridging the gap between these two classes of materials, an ideal polymeric material would combine processability with long-term mechanical stability. In the past decades, significant attention has been directed to designing reprocessable elastomers with enhanced dimensional stability, leveraging dynamic covalent bonds,^3–5^ supramolecular interactions,^6,7^ or ionic associations.^8–11^ Among these, ion-containing polymers, especially the ones with low charge densities, i.e., ionomers, offer unique opportunities due to their ease of synthesis, tunable dynamic interactions, and potential reprocessability.^12–14^ The inter- and intrachain ionic associations significantly slow down polymer dynamics at various length scales, reflected by an increase in glass transition temperature (segmental),^15,16^ an extended rubbery regime (chain strand entanglement),^17,18^ and delayed terminal flow (whole chain relaxation).^19,20^ In ionomers where ions are randomly distributed along the polymer chains, the inherent incompatibility between ionic moieties and polymer matrix results in ion multiplets or clusters with diameter of 1–3 nm and spacing of 3–10 nm.^12^ Specifically, for stress relaxation, one ionomer segment must pull out from one cluster while finding an adjacent, different one to reinsert into, during which multiple pullout and reinsertion events may occur.^21^ As a result, the terminal flow time increased by orders of magnitude. However, since the ionic association energy is still lower than the covalent counterpart, ionomers typically soften and even flow at elevated temperatures when ion-pair dissociation occurs.^22,23^ This thermoplastic property allows for thermal processing but compromises the creep resistance.

In this work, we explore a strategy for rational microstructure design in ionomers to achieve a balance between creep resistance, mechanical recoverability, and thermal reprocessability. The central hypothesis is that the mechanical integrity and creep resistance are directly related to the size and spacing of the ionic domains. Since chain exchange from one domain to another involves a combination of chain pull-out, diffusion, and reinsertion,^24–26^ increasing the ionic domain size from 1–3 nm to >10 nm raises the effective activation energy for ionic dissociation and thus drastically prolongs the chain pull-out time relative to individual ionic stickers, ion pairs, or small clusters.^27^ Meanwhile, increasing the spacing between ionic domains from 3–10 nm to >30 nm further extends diffusion times. In short, we envision that microphase-separated ionomers with large and well-segregated long-range ordered ionic domains will demonstrate unique mechanical stability while still allowing for rearrangements of the flexible charge-neutral domains.

Here, we chose a poly­(isobutyl acrylate)-based ionomer with tertiary ammonium chloride ionic moieties as a model system. When the ions are randomly distributed along the chain at ca. 20 mol % charge density, the ionomer is elastic at room temperature but turns into a viscous liquid above 68 °C (where storage and loss moduli are identical) due to ion dissociation.^20^ When the ionic monomers are confined into a block, the enthalpic penalty of mixing of the two blocks results in a long-range ordered structure with an ionic/neutral domain spacing of ca. 30 nm. Since chain exchange between adjacent ionic domains is substantially slowed, the diblock ionomer remains in the rubbery regime at *T* > 35 °C until degradation temperature. The material demonstrates excellent creep resistance (creep compliance power law scaling *J*(*t*) ∼ *t* ^0.14^) and recovery after stress removal (>90% after five consecutive creep-recovery cycles), comparable to commercial triblock thermoplastic elastomers.^28^ More importantly, the low *T*_g_ of the neutral block allows for reprocessing by hot pressing at 80 °C. Our work sheds light on the design of thermally reprocessable polymeric materials with creep resistance and recoverability by optimizing ionic self-assembly.

## 2 Results

### 2.1 Polymer Synthesis and Properties

All ionomers were synthesized by reversible addition–fragmentation chain-transfer (RAFT) copolymerization of the charge-neutral isobutyl acrylate (iBA) and cationic [2-(acryloyloxy)-ethyl]­trimethylammonium chloride (ATMAC) monomers, targeting a 20 mol % charge fraction, and the characteristics are summarized in Table 1. The synthesis of the random ionomer (r-100) and diblock ionomer (b-120) was outlined in our previous work.^20^ Another diblock ionomer with a longer chain length, b-220, was prepared with a different chain transfer agent 2-(Dodecylthiocarbonothioylthio)-2-methylpropionic acid (DMAT) to enable better control of polymerization kinetics (Figure 1). The *T*_g_ of r-100 is −16 °C, which is higher than that of homopolymer PiBA (−27 °C).^20^ This is because the cationic monomers that are randomly distributed along the polymer chain limit the motion of nearby PiBA segments. For both b-120 and b-220, only a single *T*_g_ at −25 °C was detected, which is attributed to the neutral PiBA block, and the *T*_g_ of the glassy charged block PATMAC (polyelectrolyte) was not attainable. The low *T*_g_ close to that of pure PiBA homopolymer (−27 °C) suggests effective phase separation between the two blocks, where the glassy block has minimal impact on the segment mobility of the neutral rubbery block.

**Figure 1:**
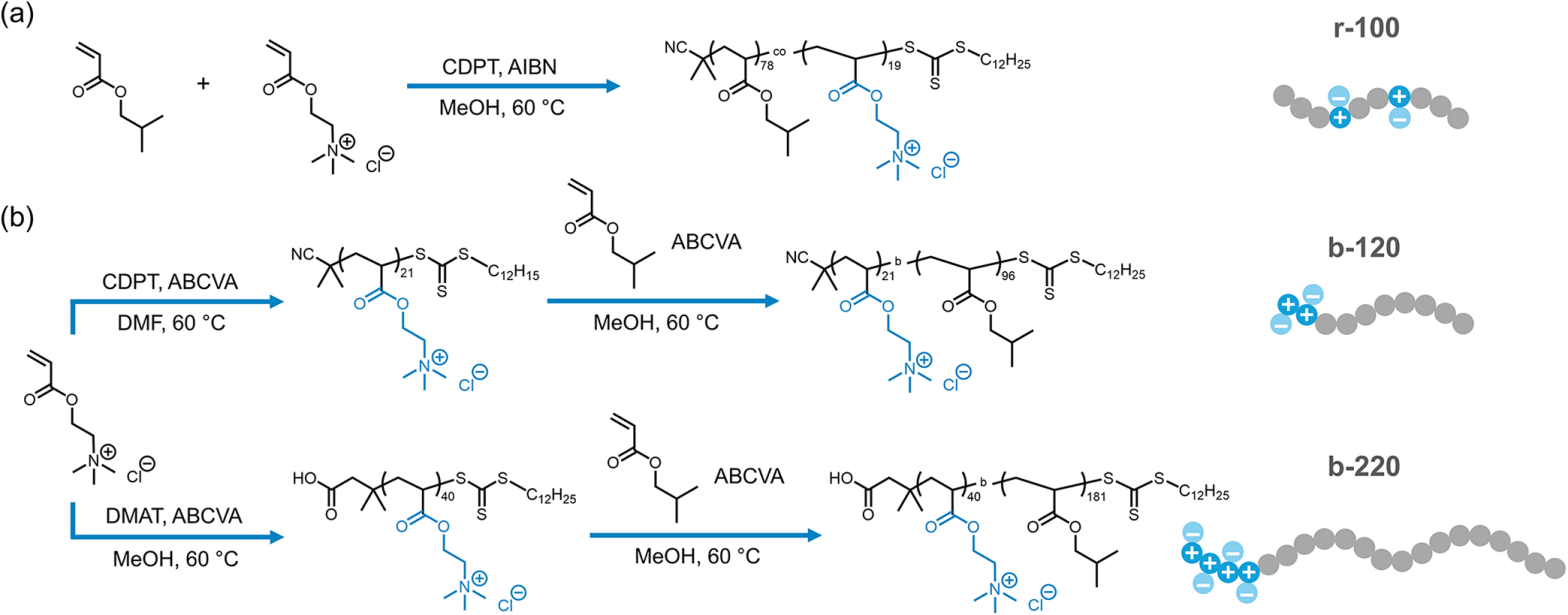
Synthesis routes of (a) random ionomer r-100 and (b) diblock ionomers b-120 and b-220. The numbers denote the total degrees of polymerization.

**Table 1:** Characteristics of Cationic Random and Block Ionomers

	charged block length (*N*_ATMAC_)^a^	neutral block length (*N*_iBA_)^a^	*N*_total_ ^a^	mole fraction (*f*_ATMAC_)	volume fraction (*f*_ATMAC_)^b^	morphology	domain spacing (*d*_10_) [nm]	*T*_g_ [°C]
r-100	19.4^c^	77.6^c^	97	0.20	0.25–0.26	LLP	3–4	–16
b-120	21.2	96.4	117.6	0.18	0.23–0.24	iHEX	30.6	–25
b-220	39.9	181.4	221.3	0.18	0.23–0.24	iHEX	50.7	–25

a Calculated by monomer conversion based on ^1^H NMR.

b Calculated based on PiBA density 1.07 g/cm^3^ and estimated ATMAC monomer density 1.17 g/cm^3^.^18^

c Charge monomers are randomly distributed along the polymer chain.

### 2.2 Viscoelasticity and Creep-Recovery Properties

Random ionomers form inter- and intrachain ionic associations that produce 1–3 nm ionic clusters.^12^ These clusters act as physical cross-links that offer mechanical integrity and substantially delay chain relaxation. For example, with 20 mol % charge, r-100 exhibits a terminal relaxation time more than 4 orders of magnitude longer than PiBA.^20^ However, as shown in Figure 2a, r-100 softens and flows above 68 °C due to accelerated ion-pair relaxation and the loss of topological constraints. By contrast, when the same number of cationic monomers is arranged in a block sequence, the resulting b-120 diblock ionomer retains elasticity over a much wider temperature window, maintaining a storage shear modulus of 0.1–0.7 MPa from room temperature to 135 °C. To assess the stress-bearing capability of this block ionomer elastomer, we conducted stress-relaxation measurements. As shown in Figure 2b, the shear modulus of r-100 decays from 6.7 × 10^7^ Pa to 9.4 × 10^5^ Pa within 10^3^ s at 35 °C, whereas b-120 remains within the same order of magnitude over the same time scale. Remarkably, even at 135 °C, only minimal relaxation was observed for b-120, indicating that the physical network remains intact, and no terminal flow occurs up to this temperature.

**Figure 2:**
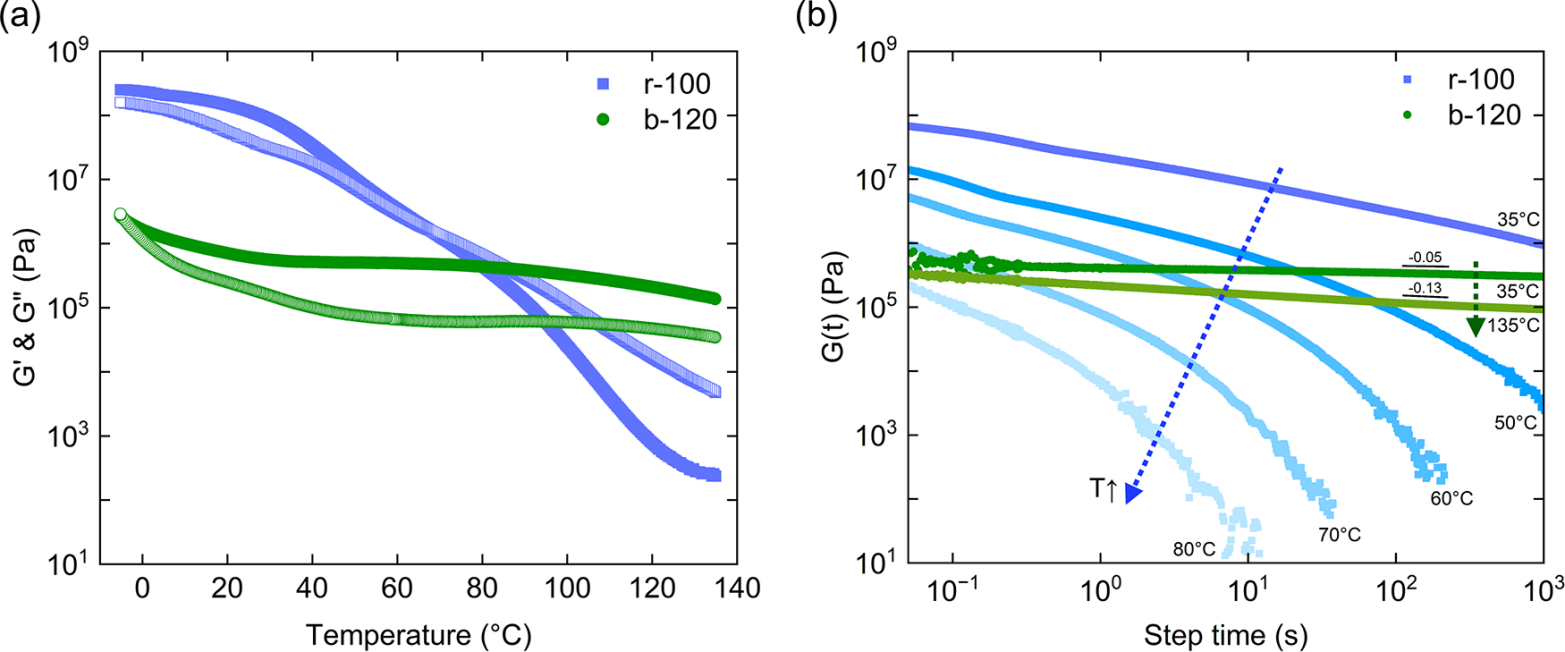
(a) Temperature sweeps of r-100 and b-120. Solid symbols represent the storage modulus (*G*’) and open symbols represent the loss modulus (*G*”). (b) Stress relaxation spectra of r-100 and b-120. r-100 relaxes as the temperature increases, while b-120 remains rubbery from room temperature to 135 °C and shows limited relaxation.

We then carried out the time–temperature superposition (tTS) mastercurve to quantify the relaxation behavior of r-100 (Figure S11). The characteristic relaxation time was around 790 s when applying the reference temperature at 35 °C. At 75 °C, the characteristic relaxation time dropped to less than 1 s, which is around 3 orders of magnitude shorter than that at 35 °C. This again justified the accelerated chain relaxation and ion-hopping event with increasing temperature and agree well with the r-100 stress-relaxation profile, where the modulus decays faster with increasing temperature. It is more complicated to quantify the characteristic relaxation of a block ionomer elastomer through stress-relaxation fitting due to the heterogeneous microstructure and a broader relaxation spectrum. As shown in Figure 2b, like many elastomers, a power law relaxation was observed

$$G\left(t\right)∼t^{-\alpha }$$ 1

where α = 0.05 at 35 °C and α = 0.13 at 135 °C. This indicates that in the experimental time window, chain relaxation is too slow to achieve steady-state flow.

To evaluate whether the elastomer will hold its shape and function over time, creep-recovery tests were carried out (Figure 3). When a constant stress of 25 Pa is applied to r-100 at 135 °C, the strain increases linearly with time and shows almost no recovery after stress removal, agreeing with its terminal behavior. In contrast, b-120 shows viscoelastic behavior in the creep regime, followed by a strain recovery of more than 85% after stress removal. To normalize the material deformation during creep (*t* < *t*_0_), we calculated creep compliance

2 $$J\left(t\right)=\frac{\gamma \left(t\right)}{\sigma_{0}}$$

where γ­(*t*) is the strain during creep, and σ_0_ is the creep stress. For r-100, *J*(*t*) increases linearly with *t*, indicating terminal flow, while for b-120, a much weaker power law scaling is observed, *J*(*t*) ∼ *t* ^0.14^, suggesting that the creep resistance is comparable to thermoplastic elastomers and remains far from achieving steady-state flow.^29^ As r-100 reaches a steady-state flow at 135 °C, the measured recoverable compliance plateau value *J*_rec_(t) represents steady-state creep compliance *J*_e_^0^

3 $$J_{\mathrm{rec}}\left(t\right)=\frac{\gamma \left(t_{f}\right)-\gamma \left(t+t_{f}\right)}{\sigma_{0}}$$

where γ­(*t*_f_) is the strain at the end of the creep test, γ­(*t* + *t*_f_) is the strain at the end of the recovery test, and σ_0_ is the creep stress. We can further calculate the steady-state relaxation time τ_ss_ = *J*_e_^0^η_0_, where τ_ss_ is around 3.14 s. The plateau value of *J*_rec_(t) at *t* = 1320 s of b-120 is almost 3 orders of magnitude smaller than r-100, due to the higher modulus of the block ionomer. However, the ratio *J*_rec_(*t*)/*J*(*t*_f_) is 0.42 in b-120, while it is almost 0 for r-100. This indicates excellent recoverability of b-120.

**Figure 3:**
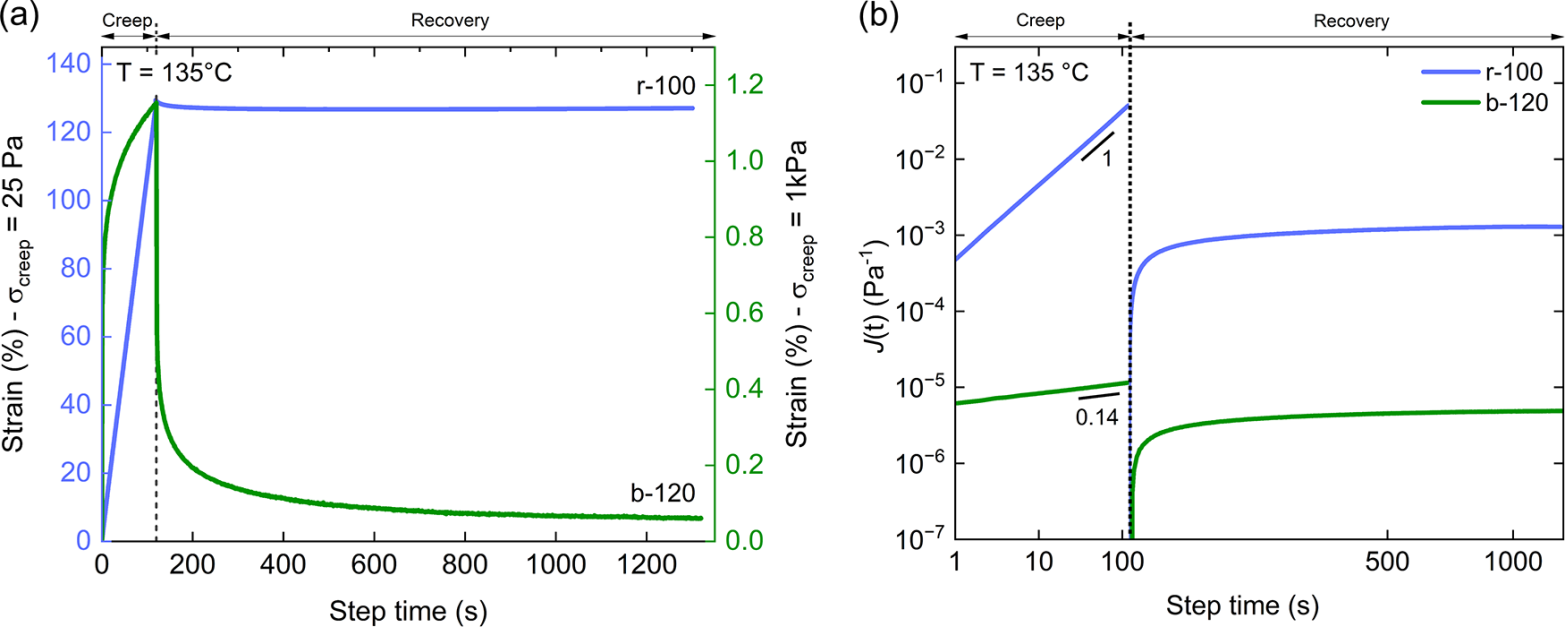
(a) Strain profiles and (b) creep compliance, *J*(*t*), and recoverable compliance, *J*_rec_(*t*), of r-100 and b-120 at 135 °C. With almost identical chain lengths but different ion distributions, r-100 reaches steady-state flow in creep and shows minimal recovery, while b-120 exhibits creep resistance and good recovery behavior.

It is worth noting that the ionic compositions and total chain lengths of r-100 and b-120 are nearly identical and remain well below the entanglement chain length (see Supporting Information for details).^30^ Thus, the pronounced enhancement in elasticity is likely attributable to the well-segregated nanostructures developed in the block architecture. The large ionic domain size and spacing effectively constrain chain pull-out and significantly slow interdomain diffusion, driving a transition from a viscoelastic liquid to an elastic solid. Next, we characterize the microstructure and illustrate the structure–property relationship in ionomer elastomers.

### 2.3 Microstructures in Diblock Ionomers

The microphase separation and morphology were characterized by atomic force microscopy (AFM) and small- and wide-angle X-ray scattering (SAXS/WAXS). In r-100 (20 mol %), the ionic groups aggregate into clusters with a diameter of 1–3 nm dispersed in a flexible PiBA matrix. The broad peak at 0.17 A^–1^ indicates that the small clusters are liquid-like packed with a spacing of ca. 3–4 nm. In b-120 (18 mol %), however, no such ion cluster was observed, and instead, multiple Bragg peaks were observed, and the peak positions indicate a long-range hexagonally packed cylindrical morphology. The domain spacing is calculated as *d*_10_ = 2π/q* = 30.6 nm, where q* is the main peak position, and thus the cylinder-to-cylinder distance $L=d_{10}/\frac{\sqrt{3}}{2}=35.3\mathrm{nm}$ . More importantly, AFM nanomechanical analysis reveals that the cylinders are softer than the continuous matrix (Figure 4b). Since the *T*_g_ of the PATMAC block is much higher than that of PiBA and thus stiffer, and the volume fraction is only ca. 24 vol %, we conjecture that an “inverse hexagonal cylindrical” (iHEX) phase is formed in b-120 (Figure 4c). Such an inverse structure has been reported in surfactant systems but is less common in polymer melts.^31–34^ Simulation work by Sing and co-workers suggests that the ion–ion correlation in diblock ionomers may result in a tilted phase diagram where an iHEX with relatively low-volume fraction of the charged block was stabilized.^35–37^ Earlier experimental work by Eisenberg and co-workers demonstrated that when the volume fraction of ionic clusters in ionomers lies between 0.6 and 0.8, the size of the clustered region will grow until continuity is established, leading to phase inversion.^38^ Russell et al. also reported inversed microphases in charge-neutral diblock copolymers where the charged block forms the continuous matrix,^34^ although the volume fraction of the charged block is larger than the ionomers we report here. Moreover, theoretical analyses by Semenov and Nyrkova suggest that nonspherical multiplets can be stabilized in ionomers, potentially contributing to enhanced mechanical properties.^39,40^ The self-assembly mechanism of the iHEX phase is beyond the scope of this paper, but we note that b-120 was dissolved in methanol before drying. Methanol can be a selective solvent to the polar PATMAC block and thus swells the charged domain, resulting in a kinetically trapped iHEX phase after solvent evaporation. However, scattering profile shows that iHEX phase persisted at 135 °C, indicating stability of this potential metastable state at working conditions (*T* < 135 °C) (Figures S13 and S14). To test the generality of the iHEX morphology, we synthesized an 18 mol % block ionomer with doubled chain length, i.e., b-220. Similarly, an iHEX morphology was observed with *d*_10_ = 50.7 nm and *L* = 58.5 nm. We note that such well-segregated block copolymer type morphologies are rare in ionomers.^12,18,41–44^ Most alkali metal sulfonate ionomers, even when adopting a blocky architecture, demonstrated micellar assembly, potentially due to the strong electrostatic cohesion of sulfonates.^18^ Moreover, the micelle size ranges from 1–5 nm, and as charge density increases, the intermicelle (or intercluster) distance decreases.^45^ The ammonium chloride ionomer model system, however, assembles into well-ordered cylinders with a spacing more than 10 times larger than that of typical ion clusters (Figure 4c). We then evaluate the chain conformation in structured b-120 and b-220. Since the PATMAC conformation is not accessible, we assume the conformation to be similar to PiBA, of which the statistical segment length *b* = 5.56 Å.^46^ For b-120, the contour length *L*_con_ = *Nl* = 36.2 nm, where *l* is the chemical bond length of two C–C bonds, and the unperturbed coil end-to-end distance is <*h* ^2^>_0_^1/2^= *N* ^1/2^ *b* = 6.03 nm. Since the PATMAC block is more rigid, we speculate the actual <*h* ^2^>_0_^1/2^ can be somewhat larger than 6.03 nm. Since the cylinder-to-cylinder distance *L* = 35.3 nm for the iHEX, the stretching ratio $\frac{L/2}{{<h^{2}>}^{1/2}}=2.9$ , indicating a highly stretched state due to the large enthalpic penalty of mixing. For b-220, the stretching ratio is even larger, ca. 3.5. The stretched chain conformation is justified by the large Flory–Huggins interaction parameter χ_eff_ between the charged and neutral monomers, and thus the segregation strength χ_eff_ *N* is substantial.

**Figure 4:**
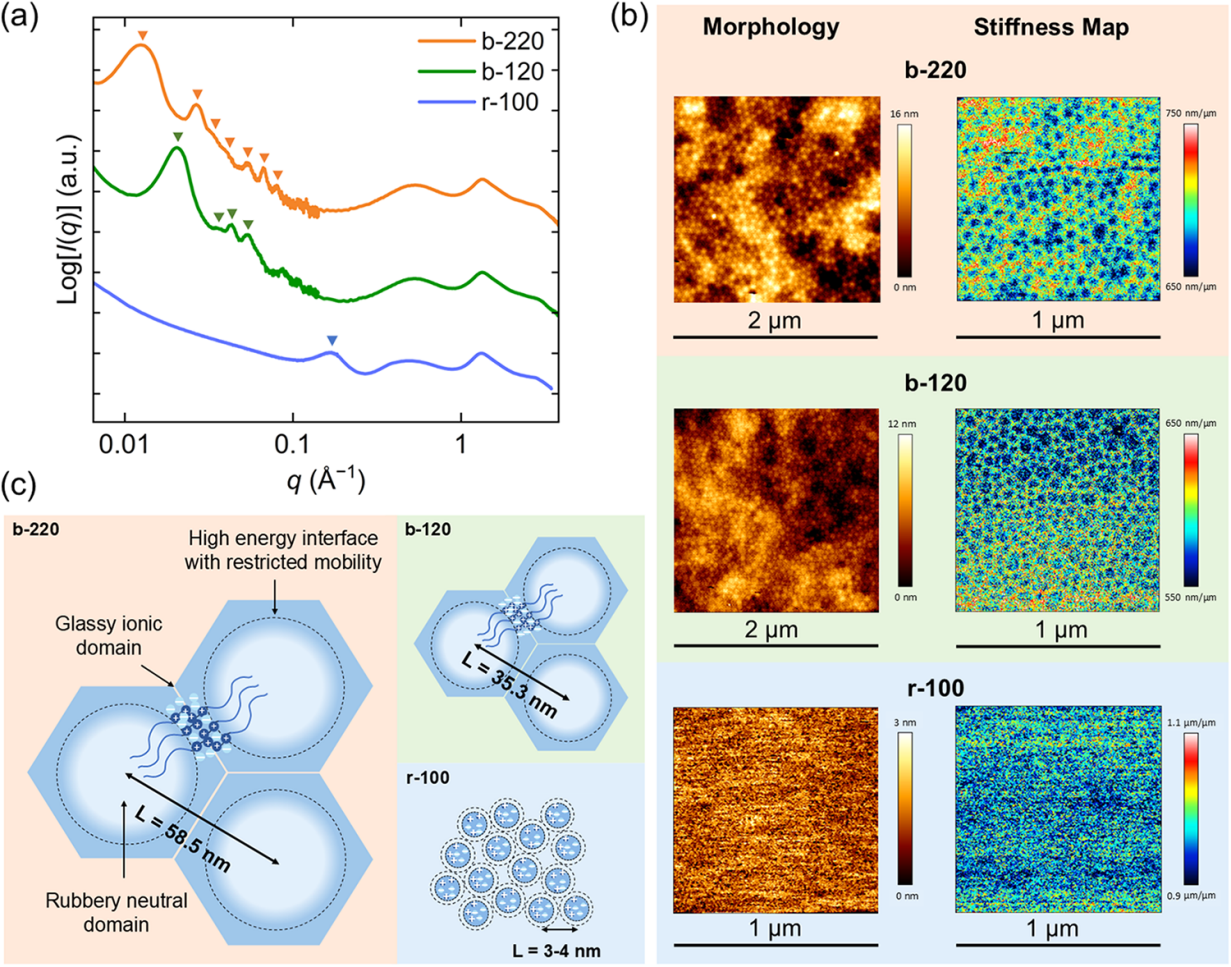
(a) SAXS/WAXS profiles suggest long-range ordering of ionic domains in a hexagonal cylinder lattice in b-220 (orange) and b-120 (green), while r-100 (blue) demonstrates liquid-like packing of ionic clusters. (b) Atomic force microscopy (AFM) morphology and stiffness mapping confirm the iHEX morphology in block ionomers where the cylinders (PiBA) are softer than the matrix (PATMAC). (c) Schematic representation of the iHEX morphology with rubbery charge-neutral domains dispersed in an ionic glassy scaffold. The spacing between ionic clusters in the random ionomer is 1 order of magnitude smaller than in the block ionomers.

### 2.4 Processability of Diblock Ionomers

Owing to the iHEX morphology, b-120 demonstrates dimensional stability under deformation. At 35 °C, the diblock ionomer recovers more than 90% of the strain even after five consecutive creep–recovery cycles. At 80 °C, however, enhanced chain relaxation reduces creep recovery and diminishes dimensional stability (Figure 5a). Therefore, 80 °C was selected as the processing temperature. The reprocessability of b-120 was assessed by breaking the sample into pieces, followed by compression molding the pieces at 80 °C. An optically transparent film was obtained within 3 min, while a bar-shaped specimen (30 × 8 × 1.59 mm^3^) formed within 30 min (Figure 5b). After remolding, no fracture was observed by optical microscopy, confirming macroscopic healing within a short time (Figure S16). After one (b-120 r1) or two (b-120 r2) reprocessing cycles, mechanical properties were characterized. Both temperature and frequency sweeps showed no deterioration in modulus relative to the pristine sample (Figure 5c,d). Moreover, stress-relaxation tests revealed negligible decay in modulus, underscoring the material’s resistance to time-dependent softening and its suitability for long-term load-bearing applications (Figure 5e).

**Figure 5:**
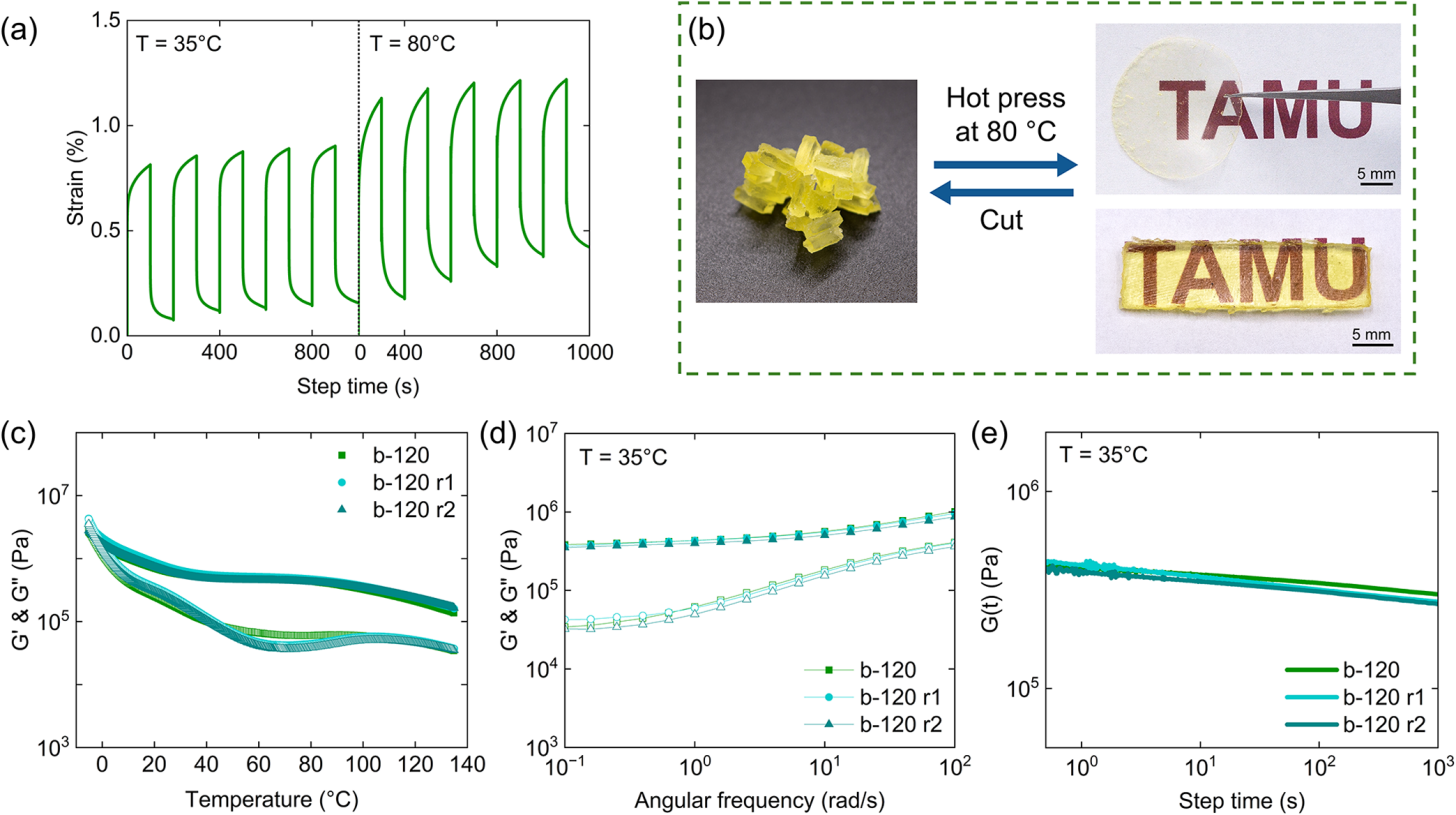
(a) Cyclic creep recovery of b-120 at 35 and 80 °C with 2 kPa stress. (b) Reprocessing b-120 via hot pressing at 80 °C. (c) Temperature ramp, (d) frequency sweep, and (e) stress relaxation of pristine b-120 and b-120 after first and second hot-pressing cycles. Solid symbols represent the storage modulus (*G*’) and open symbols represent the loss modulus (*G*”). b-120 exhibits excellent reprocessability while maintaining its mechanical integrity.

## 3 Discussion

In block copolymers with rubbery and glassy blocks, the microphase-separated morphology, whether lamellae, cylinders, or spheres, strongly influences stress relaxation by controlling chain confinement and connectivity. Unlike ABA triblocks such as polystyrene-*b*-polyisoprene-*b*-polystyrene (SIS), where midblocks bridge two glassy domains, our diblock ionomer (b-120 and b-220) has only one anchoring point per chain. The rubbery PiBA block thus extends into the soft phase as brush-like strands. Despite this architecture, the diblock ionomer exhibits remarkable elasticity due to two structural features: (1) the relatively low-volume fraction yet continuous ionic glassy domains (∼24 vol %) forming an iHEX morphology and (2) the highly stretched neutral PiBA blocks within ∼32 nm cylinders (Figure 4c). The former provides a rigid, load-bearing scaffold, favoring creep resistance and recoverability, while the latter underpins thermal processability.

### 3.1 Creep Resistance and Recoverability

Conventional TPEs such as SIS achieve recovery through PS end block anchoring, but long-term stress or elevated temperatures eventually allow chain pull-out and microdomain rearrangements. In contrast, the diblock ionomer maintains >85% recovery even at 80 °C, despite lacking triblock connectivity and entanglement (PiBA block is shorter than its entanglement length). This performance originates from its ordered microstructure and two kinetic barriers during chain exchange: (1) the large enthalpic penalty for chain pull-out from the glassy ionic domains, and (2) the large ionic domain size and large spacing, which substantially prolong interdomain chain diffusion times.^47–49^ In diblock ionomer, the rubbery PiBA block (*T*_g_ = −25 °C) forms cylinders (∼76 vol %) embedded in a continuous glassy PATMAC (*T*_g_ > 200 °C) matrix (∼24 vol %). As shown in Figure 6, in the iHEX lattice the diblock chain diffusion is anisotropic. Along the cylinder axis (z direction), the copolymer should move freely and thus the diffusion coefficient should follow Rouse dynamics.

$$D_{z}∼D_{\mathrm{Rouse}}$$ 4

**Figure 6:**
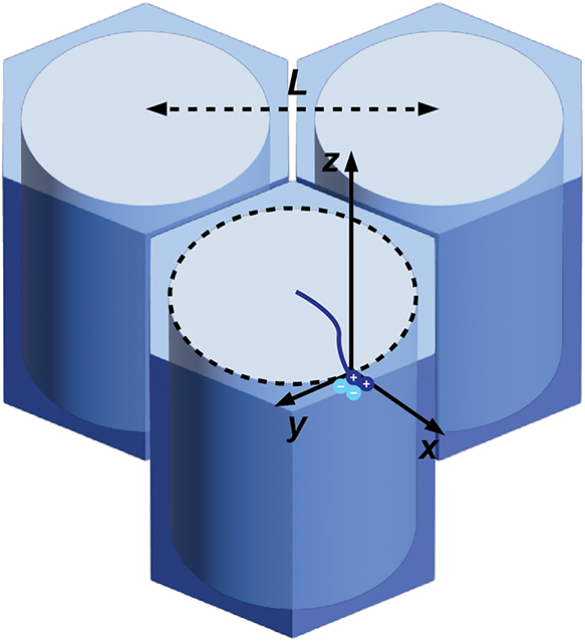
Schematic representation of the iHEX phase in b-120 and b-220. Chain diffusion along the *z* axis follows Rouse dynamics but is substantially limited in *x* and *y* directions.

Note that the Rouse time of the block copolymer is primarily dependent on the Rouse time of the charged block, which is still likely to be long. However, since the charged block is rather short and is confined in a narrow interface, the relaxation could be faster than that in a pure PATMAC homopolymer.

In directions perpendicular to the cylinder axis (*x* and *y* directions), the A-B diblock has to pull its A block into the B-rich domain and is thus enthalpically unfavored. The energy barrier to interdomain motion should scale as *αfχ*_eff_ *N*

$$D_{x}=D_{y}∼D_{\mathrm{Rouse}}\exp\left(-\alpha f\chi_{\mathrm{eff}}N\right)$$ 5

where α is a constant of order unity, *f* is the volume fraction of the charged block, and thus *D*_*x*_ and *D*_*y*_ are much slower than *D*_*z*_.^49^ The interdomain diffusion time was then calculated.

$$\tau_{\mathrm{diff}}∼L^{2}/D_{x}$$ 6

The above analysis justifies the fast dynamics in random ionomers compared with block ionomers. First, the interion cluster diffusion coefficient is much faster in random ionomers since the energy barrier is much smaller than *αfχ*_eff_ *N*. Second, the intercluster spacing in random ionomers (3 and 4 nm) is much shorter than in block ionomers (i.e., 35.3 and 58.5 nm for b-120 and b-220, respectively). As a result, τ_diff_ is orders of magnitude longer in block ionomers, resulting in elasticity across a broad temperature range. This structural feature overcomes the softening observed in conventional random ionomers at elevated temperatures due to ion dissociation. It is worth noting that previous attempts to create blocky ionomers were mostly focused on alkali metal sulfonates. Instead of forming long-range ordered block copolymer type morphologies (e.g., HEX and LAM), the sulfonate ions tend to aggregate into clusters with liquid-like packing with 3–4 nm correlation length. Thus, dynamics resemble those of TPEs, compromising creep resistance.

One should note that the block ionomer exhibits comparable, if not better, creep resistance, and enhanced recovery behavior compared to some vitrimer systems, where the network topology can switch by associative dynamic covalent bonds.^3^ In vitrimers, microphase-separated block copolymers have also been shown to suppress strand diffusion, thereby improving resistance to macroscopic deformation.^4,50–55^ By contrast, in ionomers, the blocky architecture is not just beneficial but essential for establishing elasticity: without well-defined nanostructures, the random copolymer counterpart behaves as a thermoplastic lacking thermal stability. We attribute the superior creep resistance and recovery of the diblock ionomer to two key factors: (1) the rigid, continuous PATMAC phase formation due to unfavorable enthalpic interaction that prevents chain slippage and (2) slow chain diffusion and chain exchange between ionic domains. Incorporating entanglements into PiBA or designing a PATMAC–PiBA–PATMAC triblock could further enhance elasticity and recoverability.

### 3.2 Reprocessability

Unlike conventional TPE and vitrimers, which require high temperature compression molding (e.g., at least 20–30 °C above the *T*_g_ of PS for SIS, SBS and SEBS^56^) and/or long reprocessing time, the diblock ionomer can be processed at 80 °C, well below the PATMAC *T*_g_, within a short time frame. This is because the short, unentangled PiBA chains within the rubbery cylinders can relax, especially in regions far from the glassy–rubbery interfaces. In other words, although chain exchange kinetics are slow and full terminal relaxation is inaccessible on experimental time scales, “pre-terminal” stress relaxation is possible. First, Rouse modes of the PiBA block within its domain may enable block copolymer junctions to move laterally without leaving the interface, leading to domain breathing and power-law stress decay (Figure 2b). More importantly, in the iHEX morphology, the cylindrical soft domains (PiBA) are relatively large (32.3 nm diameter) and closely spaced (center-to-center spacing ∼35.3 nm) (Figure 4c). The glassy PATMAC layers separating adjacent cylinders can be as thin as 3 nm. The glass transition temperature (*T*_g_) within such confined interfaces is likely reduced relative to the bulk value. Register and Priestley reported that *T*_g_ near the A–B diblock junction is lower than the bulk *T*_g_ of the glassy block,^57^ consistent with earlier findings on self-concentration effects.^58^ Similar conclusions were reported by Schweizer and Simmons.^59^ Consequently, chain mobility within these ultrathin glassy domains may be enhanced, reducing the barrier for interdomain diffusion and enhancing malleability. This allows block ionomers to retain good creep resistance behavior and exhibit superior reprocessability at the same time. We also note here that the compression molding is inherently nonlinear since (1) the strain rate can vary by orders of magnitude across the cavity and (2) the geometry changes dynamically; as the gap thickness decreases, a strong increase in velocity gradients is expected. Thus, although the block ionomer cannot achieve terminal flow within the experimental time scale during the small-amplitude oscillatory shear (SAOS) test, we may expect that during compression molding, intermixing between domains (chain slipping, pull-out and reinsertion) is still possible under such a large pressure and local strain rate, so the material can yield, deform, and reshape.^60^ Similar trends of ionic network materials reprocessing via compression molding were also reported by Poon et al., van Lange et al., and Cai et al.^9,61,62^ Consequently, upon deformation or cutting, compression at 80 °C (above the *T*_g_ of PiBA but well below that of PATMAC) allows the rubbery cylinders to gradually flow around the stationary glassy scaffold, re-establish interfacial adhesion, and recover mechanical integrity.

## 4 Conclusions

We demonstrate that diblock ionomers achieve an unusual combination of creep resistance, recoverability, and reprocessability despite lacking triblock connectivity and entanglement. The key lies in their long-range ordered iHEX morphology: continuous ionic glassy domains form a rigid scaffold that resists flow, while highly stretched PiBA cylinders provide local chain mobility. Together, these features substantially prolong relaxation times, suppress stress dissipation, and enable >90% recovery after five consecutive creep cycles. Unlike conventional TPEs, diblock ionomers can be processed at 80 °C, well below the *T*_g_ of its ionic phase, because rubbery domains flow around the stationary thin scaffold. This design principle, linking domain continuity and spacing to macroscopic dynamics, offers a pathway to ion-containing polymers that combine mechanical robustness with thermal processability. Further enhancement of nonlinear elasticity can be achieved by creating entanglements into PiBA or designing a PATMAC–PiBA–PATMAC triblock to improve interdomain connectivity while maintaining reprocessability. Looking ahead, we envision this framework as a broadly applicable design principle for engineering nanostructured dynamic polymers with enhanced strength, recoverability, and long-term sustainability. The iHEX morphology with continuous ionic channels may demonstrate superior ion transport properties and thus show potential in energy applications.^63^

## 5 Materials and Methods

### 5.1 Synthesis of Random and Block Ionomers

The materials and preparation process of cationic random ionomer (r-100) and block ionomer (b-120) were outlined in our previous work.^20^ b-220 was prepared by two sequential RAFT polymerizations. Starting from the cationic block, a molar ratio of ATMAC:DMAT:ABCVA = 40:1:0.2 was dissolved in MeOH and reacted for 24 h at 60 °C after N_2_ purge. After the reaction, the polymer solution was dried, redissolved in IPA, cooled down in a dry ice bath, and then centrifuged to remove residual monomers. Subsequently, a molar ratio of ATMAC:iBA:ABCVA = 40:160:0.5 was dissolved in MeOH. The polymerization conditions were identical with those of the cationic block.

### 5.2 Polymer Characterization and Thermal Analysis

The degree of polymerization (DP) and charge fraction were analyzed using proton nuclear magnetic resonance (^1^H NMR) spectroscopy on a Bruker Avance Neo 400 Hz spectrometer with methanol-d4 (99.8 atom % D) as the solvent. ^1^H NMR results and calculations are listed in the Supporting Information. The glass transition temperature (*T*_g_) was determined by a TA Instruments DSC 2500 with Tzero hermetic pan-lid assemblies. The measurement was conducted in two heating and one cooling cycles with 10 °C/min heating and 20 °C/min cooling.

### 5.3 Mechanical Characterization

TA Instruments DHR-2 and AR-G2 rheometers with an 8 mm parallel plate were used for small-amplitude oscillatory shear measurement. Temperature ramp tests were conducted between −5 and 135 °C with angular frequency at 10 rad/s and 1% strain to determine the linear viscoelasticity of r-100, b-120, b-120 r1, and b-120 r2. The stress relaxation was measured with 2% strain for r-100 at 35 °C and 50 ∼80 °C, and with 1% strain for b-120 at 35 or 135 °C. Creep-recovery measurements were performed at 135 °C, with 25 Pa stress for r-100, and 1 kPa stress for b-120. To represent the processability, a cyclic creep-recovery test was performed with 2 kPa stress with 100 s creep and 100 s recovery at 35 and 80 °C for 5 cycles. The frequency sweep was measured with 1% strain for r-100 and 0.5% strain for b-120.

### 5.4 X-ray Scattering

Small- and wide-angle X-ray scattering (SAXS/WAXS) spectra for b-120 and b-220 were obtained from Xenocs Xeuss 3.0 with a Cu Kα radiation source (λ = 1.542 Å) at Texas A&M University Soft Matter Facility. The sample-to-detector distance was 900 mm for SAXS and 45 mm for WAXS. The measurement was conducted with 10 and 5 min exposure time for SAXS and WAXS, respectively, at room temperature, 75 °C, and 135 °C. The two-dimensional patterns were recorded using a Dectris Eiger 2R 1M-pixel 2D detector to further obtain one-dimensional profiles. SAXS/WAXS spectra for r-100 were obtained from the National Synchrotron Light Source II, beamline 11-BM at Brookhaven National Laboratory, with 13.5 keV beam energy. The tests were conducted at a 5 m sample-to-detector distance for SAXS and 0.26 m for WAXS. All samples were loaded in washers and sealed with Kapton tape.

### 5.5 Atomic Force Microscopy (AFM)

AFM nanomechanical characterization of the polymer samples was performed in ambient air using the PeakForce Quantitative Nanomechanical Mapping (PFQNM) mode on an AFM system (NanoWizard 4XP, Bruker, MA). A high-resolution imaging probe (PEAKFORCE-HIRS-F-A, Bruker, MA, USA) with a nominal spring constant of 0.35 N/m and a tip radius of 1 nm was used to optimize morphological imaging. The deflection sensitivity was calibrated on a flat glass slide surface, and the spring constant was determined using the thermal tuning method prior to data acquisition. During measurements, a set point of 5 nm was maintained, with a tapping frequency of 1 kHz and a scan resolution of 256 × 256 pixels. Local stiffness was evaluated by generating slope-fit maps from the retracted segments of the force–distance curves. All data processing and quantitative analyses were performed using JPK Data Processing software (Version 8.0.111, Bruker, MA).

## Supplementary Material



## Notes

Synthetic procedures, NMR spectra, SAOS, SAXS, DSC, calculation details, and fracture healing pictures. (PDF)
