# Supplementary material for: Rational Design of Ionomer Microstructures for Thermally Reprocessable Materials with Creep Resistance and Recoverability
Source: JACS Au. 2025 Nov 29;5(12):6324–33. doi: 10.1021/jacsau.5c01317 (PMC12728631; doi:10.1021/jacsau.5c01317)
Supplement: Supplementary file 1 [file au5c01317_si_001.pdf]

Supporting information for

Rational Design of Ionomer Microstructures for Thermally Reprocessable Materials with Creep Resistance and Recoverability

Chia-Chi Tsai,[1] Hanwen Fan,[2] Yuxiao Zhou,[2] and Shuyi Xie*[1]

[1]Department of Chemical Engineering, Texas A&M University, College Station, TX 77843, United States

[2]Department of Mechanical Engineering, Texas A&M University, College Station, TX 77843, United States

*E-mail: shuyixie@tamu.edu

Materials and Synthesis (detailed)

The synthesis routes of most of the polymers described here were detailed in our previous paper.[1] The chain transfer agents 2-(dodecylthiocarbonothioylthio)-2-methylpropionic acid (DMAT) and 2-propanol (iPA, 99.9%, suitable for HPLC) were purchased from Sigma-Aldrich and used as is. In this work, all polymers were synthesized targeting a 20 mol% charge fraction. The copolymers were named according to monomer distribution ("r" stands for random and "b" stands for block) and degree of polymerization (DP). The preparation process of random ionomer poly[(isobutyl acrylate)-co-(2-acryloyloxy ethyl trimethylammonium chloride)] (r -100) and block ionomer poly[(2-acryloyloxy ethyl trimethylammonium chloride)-block-(isobutyl acrylate)] (b-120) were described in our previous work.[1]

- **Preparation of poly[(2-Acryloyloxy ethyl trimethylammonium chloride)-*block*-(isobutyl acrylate)] with a target DP of 200 (b-220)**

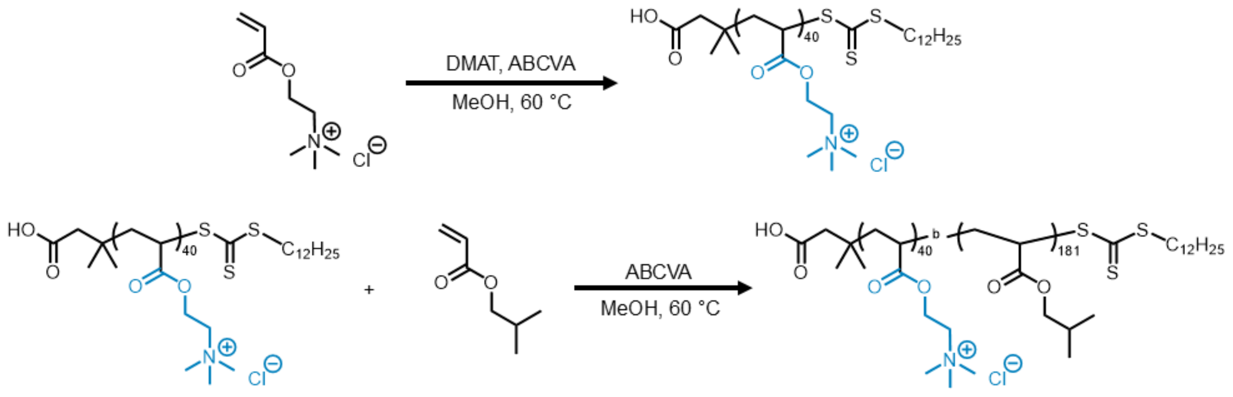

The block ionomer with a target DP of 200 (b-220) was prepared in two sequential RAFT polymerizations. First, the cationic ATMAC block with a target DP of 40 was synthesized, forming the PATMAC MacroCTA. The monomer ATMAC (5 g, 25.82 mmol), CTA DMAT (0.235 g, 0.645 mmol, from Sigma-Aldrich), and initiator ABCVA (36 mg, 0.129 mmol) were dissolved in 11 mL

of MeOH and reacted for 24 hours at 60 °C after N_2 purge. After the reaction, the polymer solution was dried by a rotary evaporator and redissolved in isopropanol (IPA). The solution was then cooled in a dry ice/IPA bath, allowing the polymer solution to phase separate, and subsequently centrifuged. The supernatant phase with residual monomers was removed, and the polymer phase was dried in a vacuum oven at 30 °C for several days. Subsequently, the MacroCTA was reinitiated where a neutral block of isobutyl acrylate (iBA) was grown. Targeting a 20 mol% charge fraction, desired amounts of the MacroCTA, monomer, and initiator with a molar ratio of ATMAC : iBA : ABCVA = 40:160:0.5 were mixed in methanol. The polymerization conditions were identical to those of the first block (PATMAC MacroCTA).

- **Materials Characterization and Thermal Analysis (detailed)**

The degree of polymerization (DP) and charge fraction of all materials were analyzed using proton nuclear magnetic resonance (^{1}H NMR) spectroscopy on a Bruker Avance Neo 400 Hz spectrometer with methanol-d4 (99.8 atom %D) as the solvent. The experiment was conducted in 16 scans with 15-second delay relaxation time at 25 °C. ^{1}H NMR results and calculations are listed in a later section. Glass transition temperature (T_g) was determined by TA Instruments DSC 2500 with around 4-6 mg material loaded in a Tzero hermetic pan-lid assembly. All measurements were conducted in two heating cycles and one cooling cycle with a ramping rate of 10 °C/min for heating and 20 °C/min for cooling for all samples. To remove the thermal history, the first heating cycle ramped up to 180 °C with a 5-minute isothermal step, and the first cooling cycle ramped down to −90 °C. The second heating cycle ramped up to 180 °C where the T_g was determined.

^{1}H-NMR

^{1}H-NMR spectra were analyzed to determine the charge density and DP of the materials. For r-100, b-120 and b-220, the peak at 0.9~1.0 ppm is assigned as six protons of the two methyl groups from the isobutyl acrylate (iBA) and used as a reference to calculate charge density and DP. The peak at 3.8~3.9 ppm is identified as the overlap of (1) two protons on the carbon directly adjacent to the ester oxygen from iBA, and (2) two protons on the carbon adjacent to nitrogen. The peak at 4.5~4.6 ppm is identified as two protons on the carbon directly adjacent to the ester oxygen from ATMAC. For PATMAC MacroCTA, the peak at 0.9~1.0 ppm is assigned as three protons of the end methyl group from chain transfer agents and used as a reference for charge density and DP calculation. The peaks at 3.8~3.9 ppm and 4.5~4.6 ppm are identified as two protons on the carbon adjacent to nitrogen and two protons on the carbon directly adjacent to the ester oxygen from ATMAC, respectively.

Figures S1 and S2 show the ^{1}H-NMR spectra of r-100 before and after dialysis. With the six protons (peak a) set as the reference, it shows that the integral of the monomer peak (between 5.8~6.4 ppm) is 0.09, suggesting that the conversion is 97%. Based on the conversion, the estimated DP is derived based on equation S1. The DP of r-100 is 97.

$$DP = \frac{[Monomer]_0}{[RAFT\ agent]_0} * Conversion \qquad (S1)$$

Again, taking six protons (peak a) as the reference, the integral of peak c (4.5~4.6 ppm) shows 0.50, meaning that 0.25 ATMAC monomer is detected per iBA unit. As a result, the charge fraction of r-100 is $\frac{0.25}{1+0.25} = 20\%$.

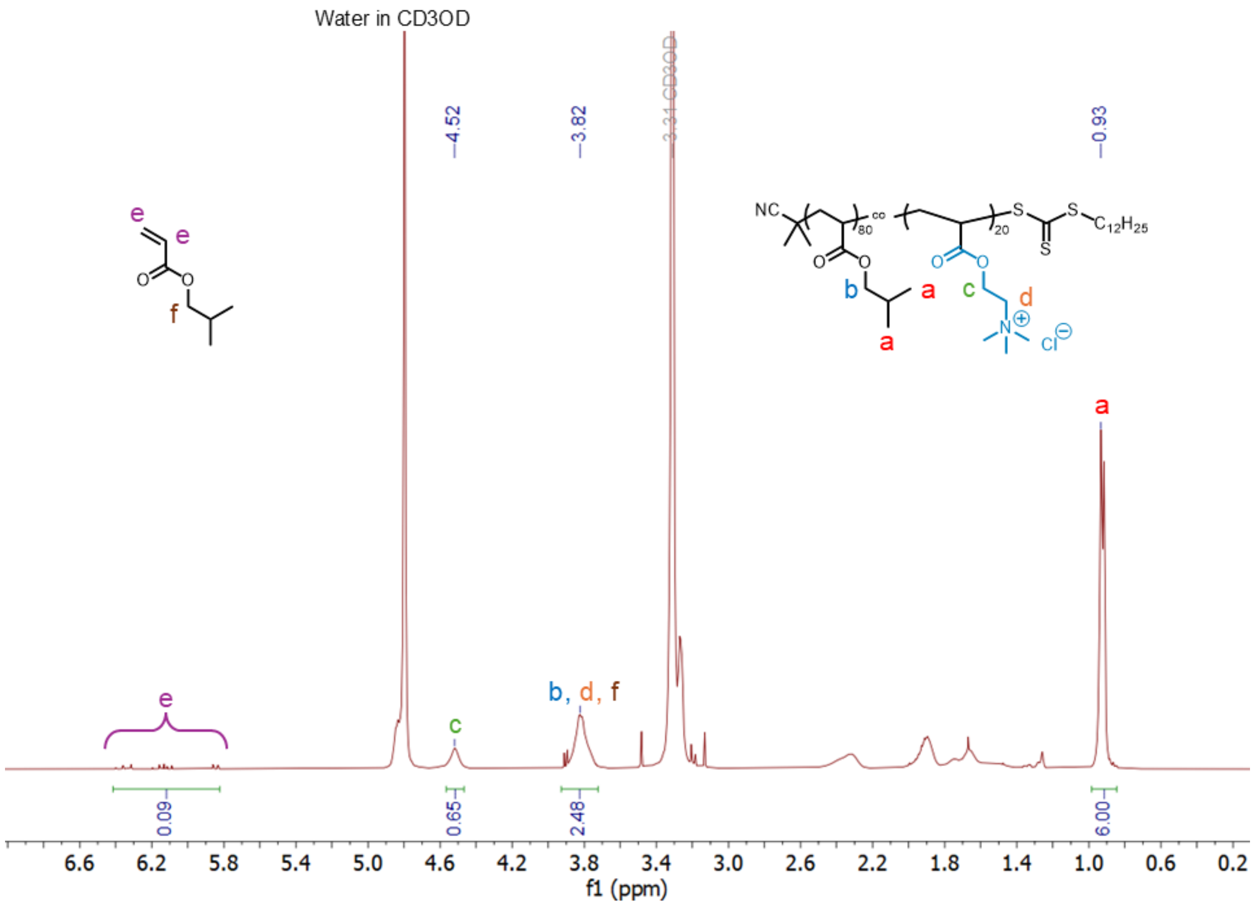

Figure S1. ^{1}H NMR spectrum of poly[(isobutyl acrylate)-co-(2-acryloyloxy ethyl trimethylammonium chloride)], r-100, before dialysis.

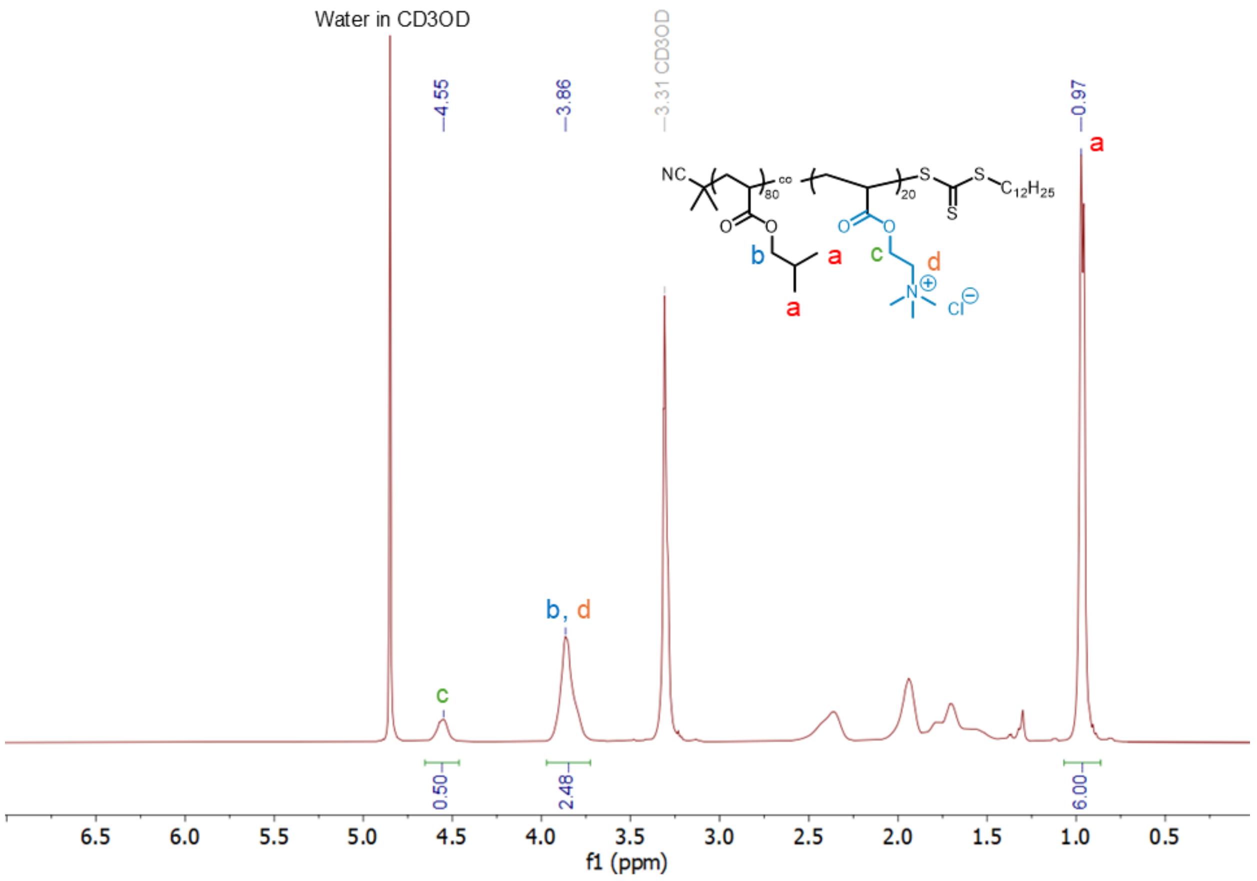

Figure S2. ^{1}H NMR spectrum of poly[(isobutyl acrylate)-co-(2-acryloyloxy ethyl trimethylammonium chloride)], r-100, after dialysis.

The ^{1}H-NMR spectrum of PATMAC MacroCTA with a target DP of 20 is reported in our previous paper.[1] The spectrum suggests a DP of 21.2 for PATMAC MacroCTA. Figure S3 shows the ^{1}H-NMR spectrum of b-120. With the same peak reference and charge fraction calculation as r-100, the integral of peak c (4.5~4.6 ppm) is 0.44, suggesting that the charge fraction is $\frac{0.22}{1+0.22}$ =18%. Further, DP of b-120 is estimated based on the charge fraction and the DP of the PATMAC MacroCTA. Accordingly, the estimated DP of b-120 is $(21.2 * \frac{(1+0.22)}{0.22})$=117.6.

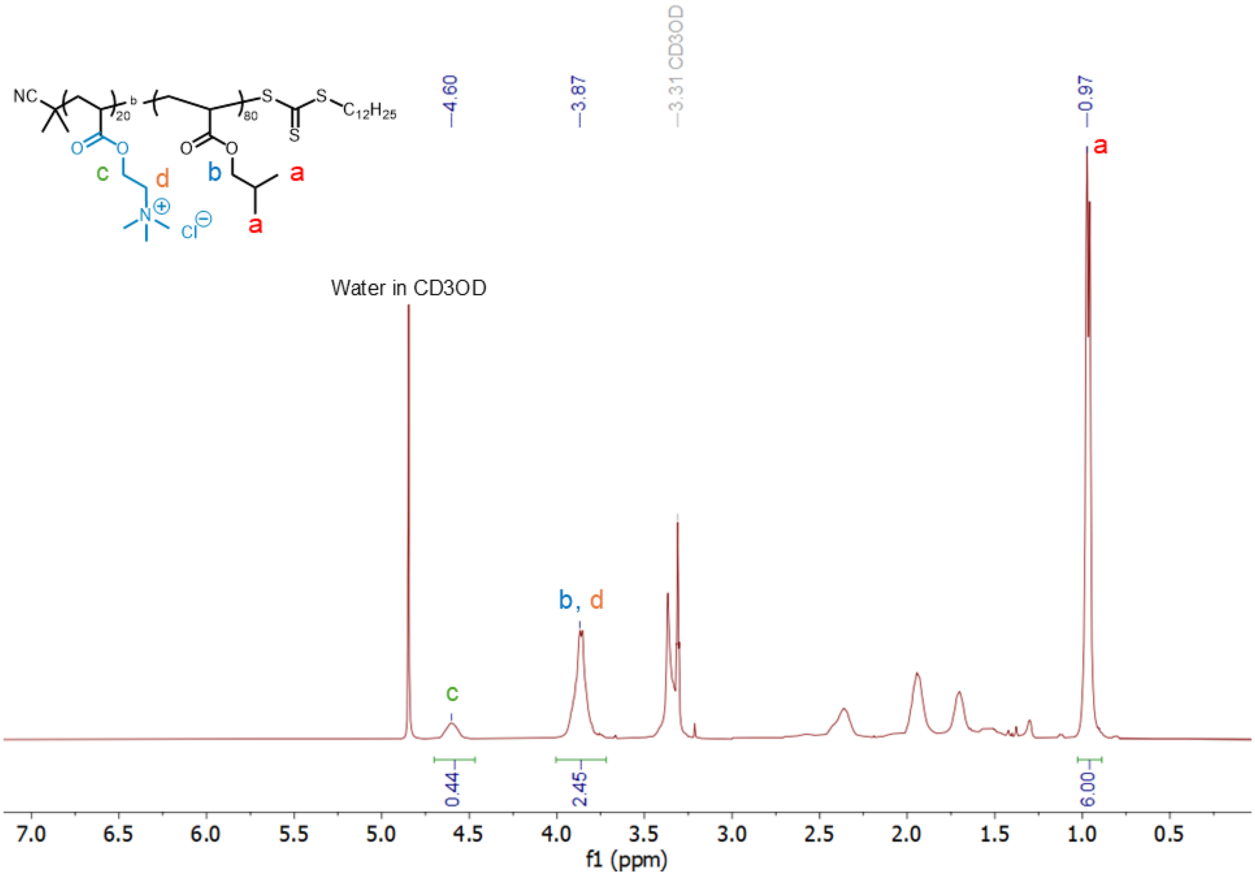

Figure S3. ^{1}H NMR spectrum of poly[(isobutyl acrylate)-b-(2-acryloyloxy ethyl trimethylammonium chloride)], b-120.

Figures S4 and S5 show ^{1}H-NMR spectra of PATMAC MacroCTA with a target DP of 40 before and after washing with IPA, respectively. Most unreacted ATMAC monomers were removed after IPA washing. Using the three protons from DMAT end methyl group as the reference, the integral of peak b (4.5~4.6 ppm) is 79.8, suggesting that the actual DP of PATMAC MacroCTA is 39.9.

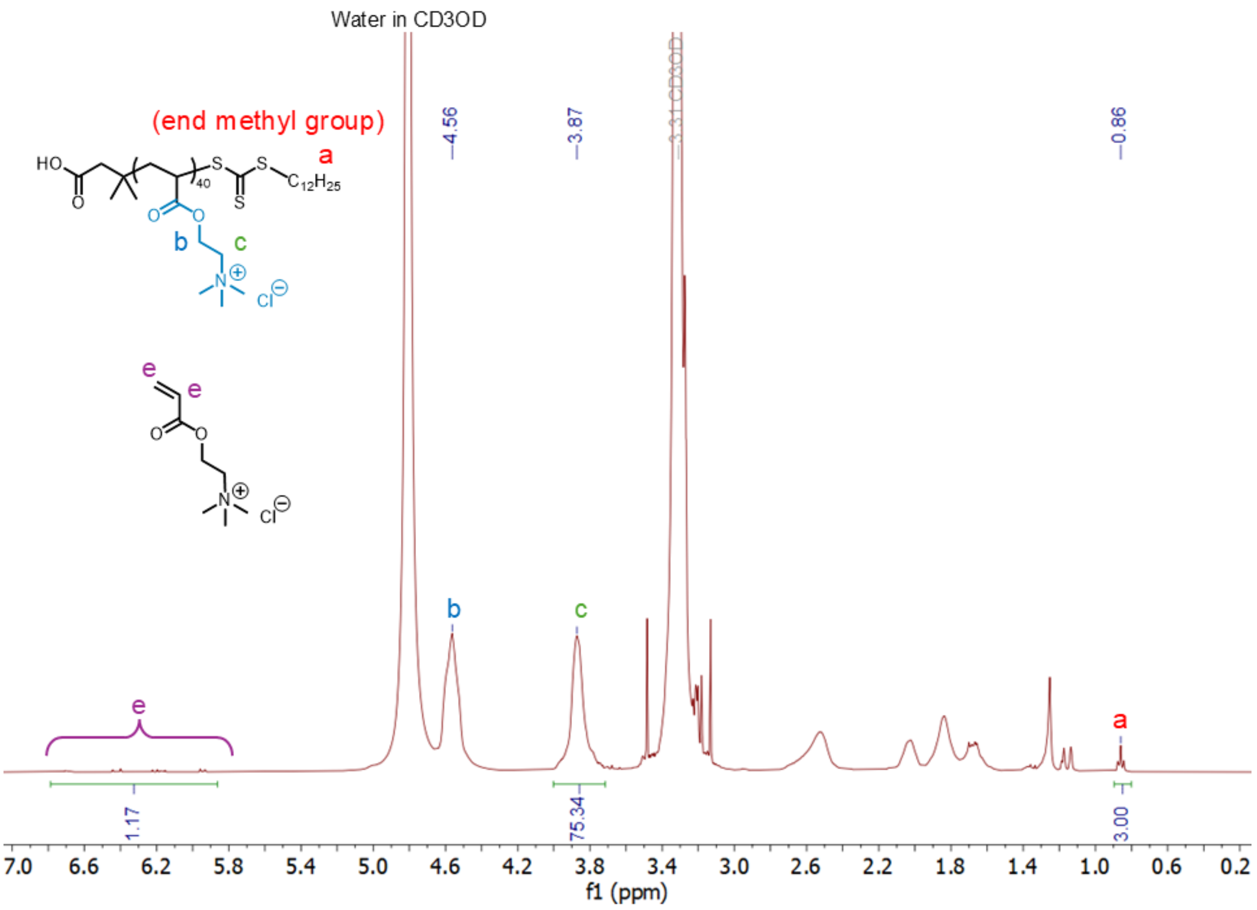

Figure S4. ^{1}H NMR spectrum of poly[(2-acryloyloxy ethyl trimethylammonium chloride)], PATMAC MacroCTA with a target DP of 40 before washing with IPA.

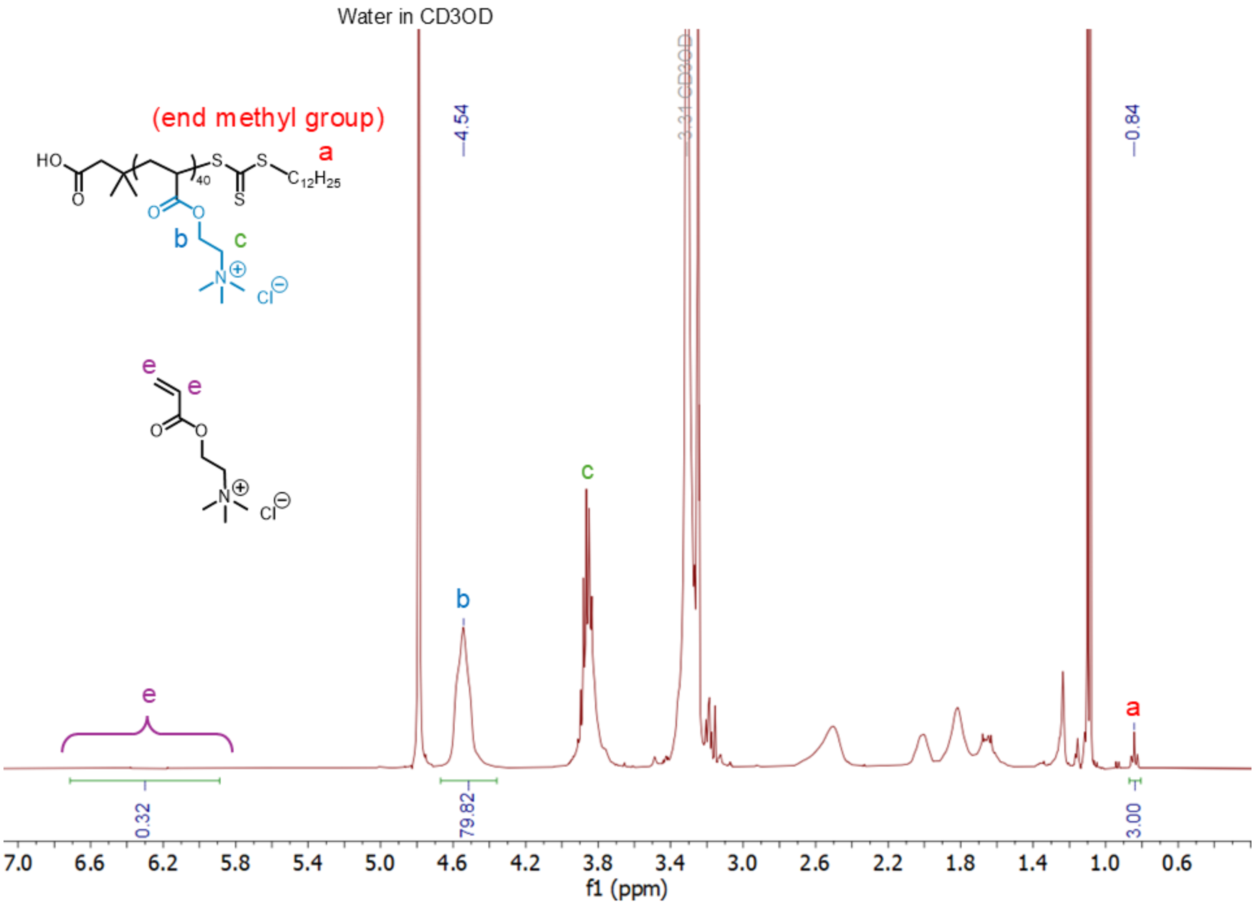

Figure S5. ^{1}H NMR spectrum of poly[(2-acryloyloxy ethyl trimethylammonium chloride)], PATMAC MacroCTA with a target DP of 40 after washing with IPA.

Figure S6 shows ^{1}H-NMR spectrum of b-220. With the same peak reference and charge fraction calculation as those used for b-120, the integral of peak c (4.5~4.6 ppm) shows 0.44, meaning the charge fraction is $\frac{0.22}{1+0.22} = 18\%$. Further, DP of b-220 is estimated from the charge fraction and DP of the PATMAC MacroCTA (Figure S5). Accordingly, the estimated DP of b-220 is $39.9 * \frac{(1+0.22)}{0.22} = 221.3$.

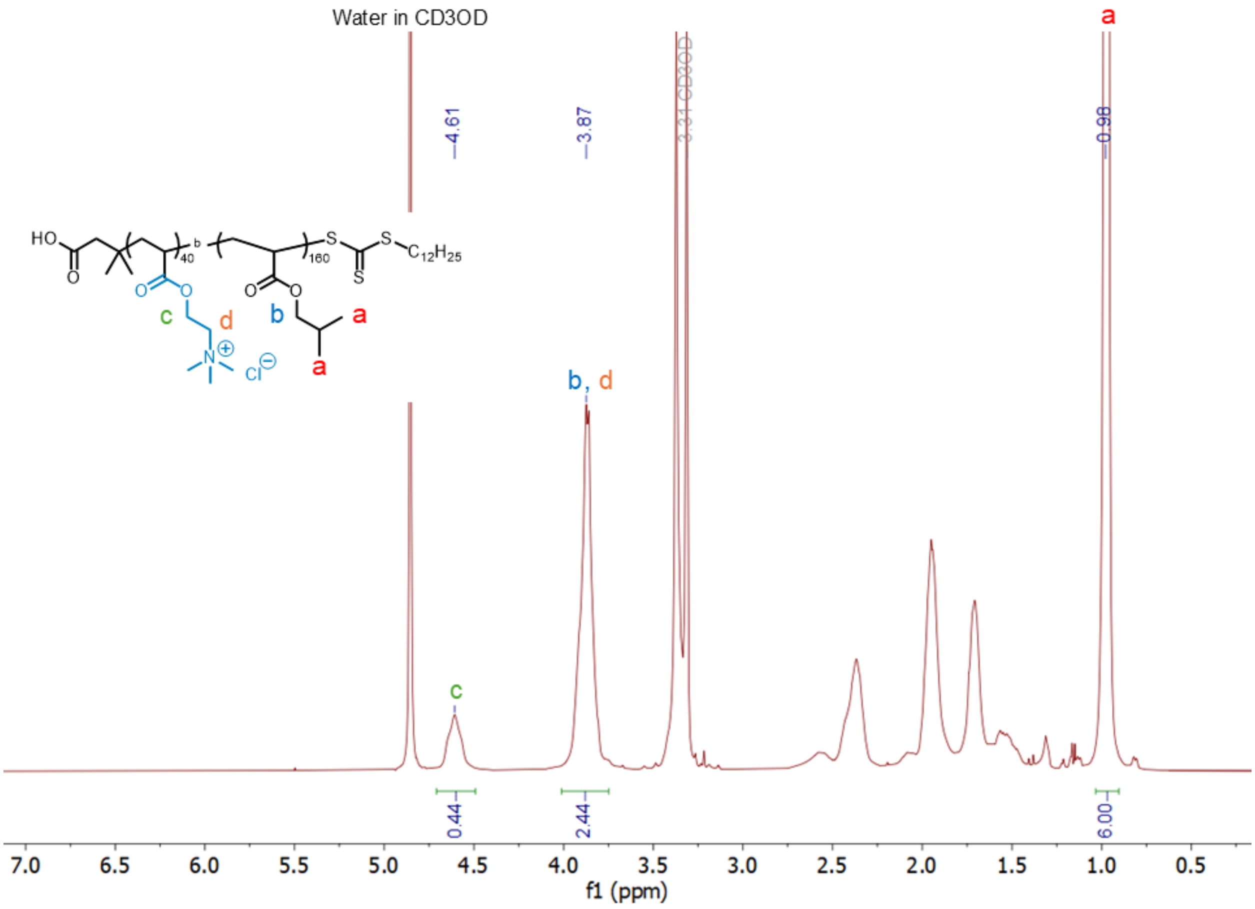

Figure S6. ^{1}H NMR spectrum of poly[(isobutyl acrylate)-b-(2-acryloyloxy ethyl trimethylammonium chloride)], b-220, after dialysis.

Entanglement Chain Length

The actual entanglement molecular weights of PiBA and diblock copolymer are not accessible. The entanglement molecular weight (M_e) for the isomer, poly(n-butyl acrylate) is 26-32 kg/mol.[1,2] To induce entanglement effect in rheology, usually the molar mass needs to be at least two times as M_e. Since the number average molecular weight of r-100 and b-120 (based on ^{1}H-NMR) is well below 50 kg/mol, we believe that both r-100 and b-120 are well below the entanglement chain length.

Glass Transition Temperature Characterization

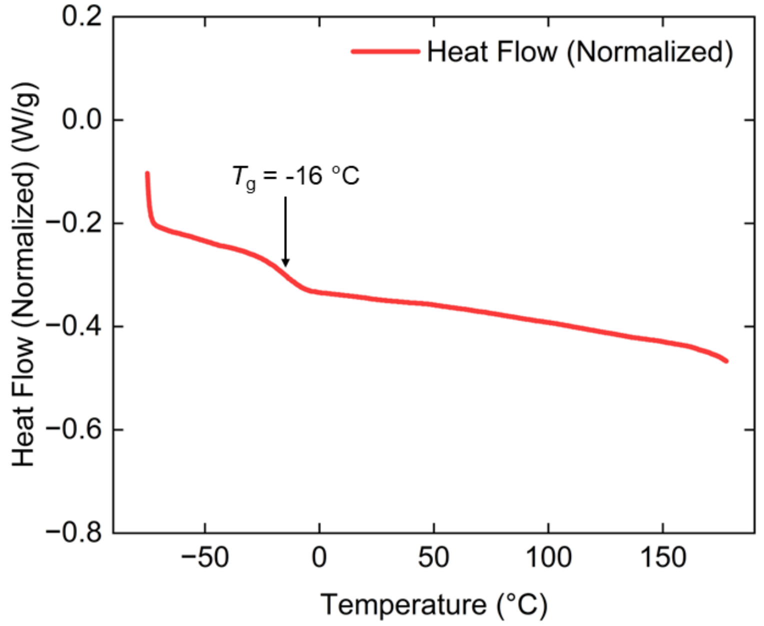

Figure S7. Differential scanning calorimetry spectrum of r-100.

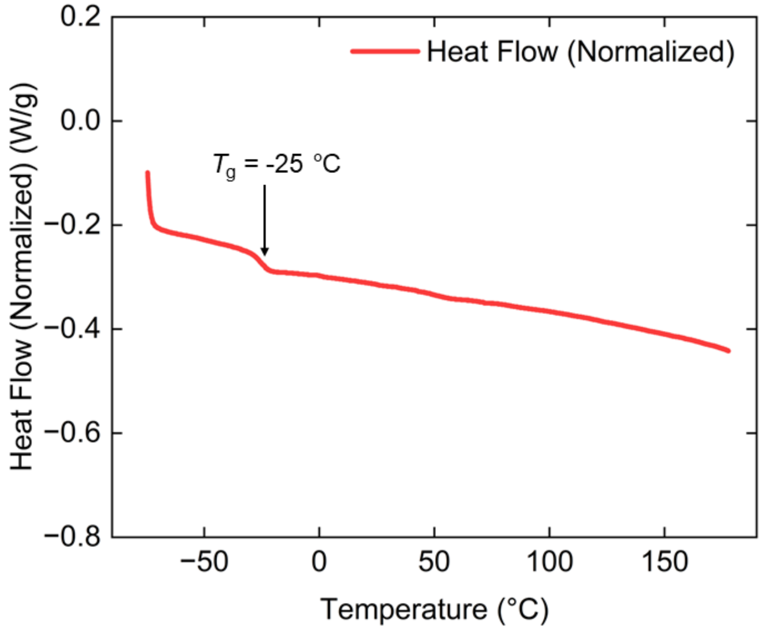

Figure S8. Differential scanning calorimetry spectrum of b-120.

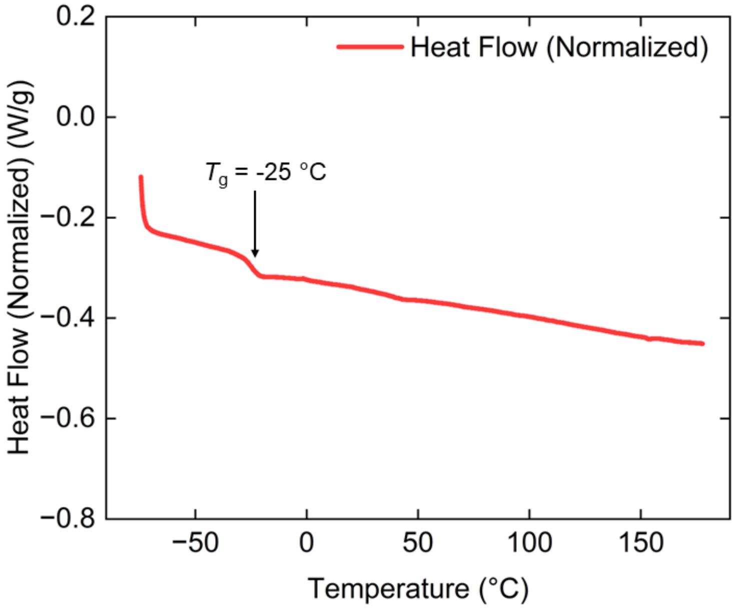

Figure S9. Differential scanning calorimetry spectrum of b-220.

Thermogravimetric analysis

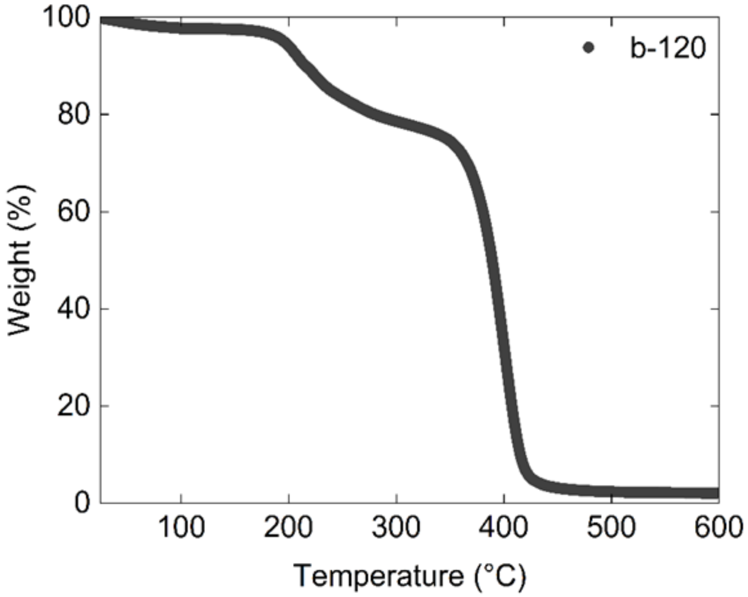

Figure S10. Thermogravimetric analysis of b-120.

Linear Viscoelasticity

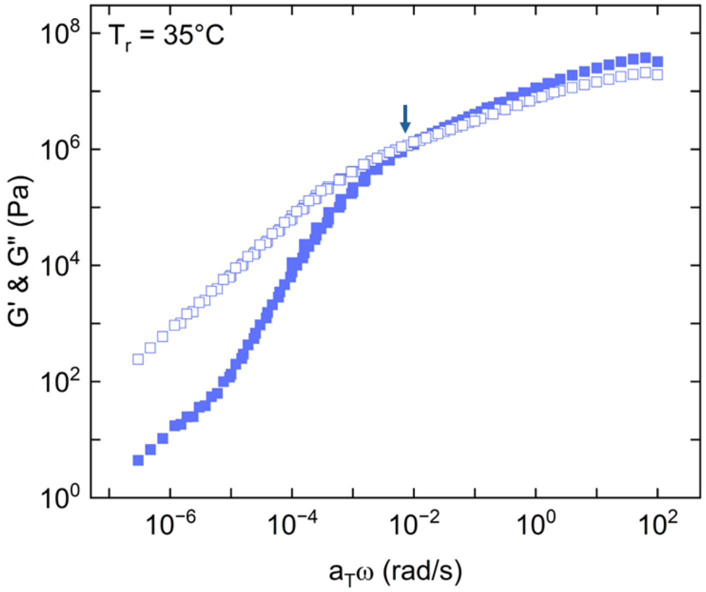

Figure S11. Time-temperature superposition mastercurve of r-100 with a reference temperature of 35 °C. Closed symbols represent the storage modulus (G') and open symbols represent the loss modulus (G").

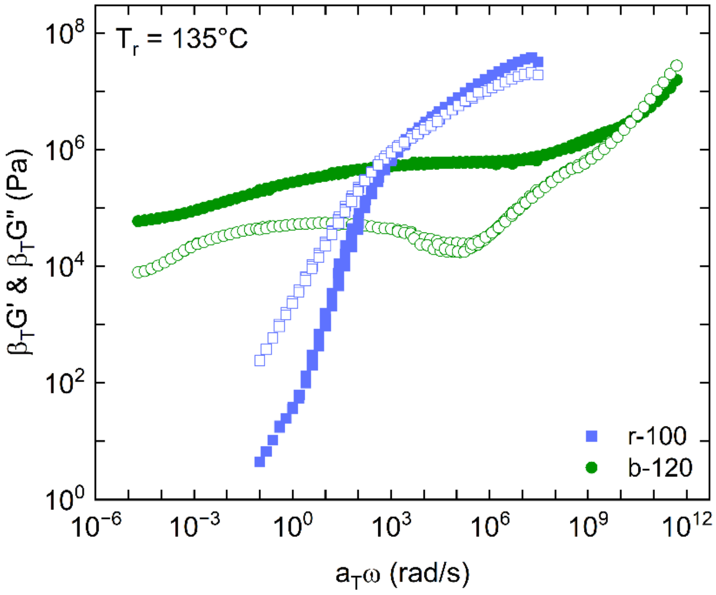

Figure S12. Time-temperature superposition mastercurves of r-100 and b-120 with a reference temperature of 135 °C. Closed symbols represent the storage modulus (G') and open symbols represent the loss modulus (G").

Note that in Figure S12, superimposing thermorheologically complex viscoelastic data of the block ionomer has questionable validity since the dynamics of the two blocks are vastly different. Nevertheless, the time-temperature superposition in a relatively narrow temperature range seems to work well and no phase transition occurs within this temperature range.[3–6]

Temperature variation small-angle X-ray scattering (SAXS)

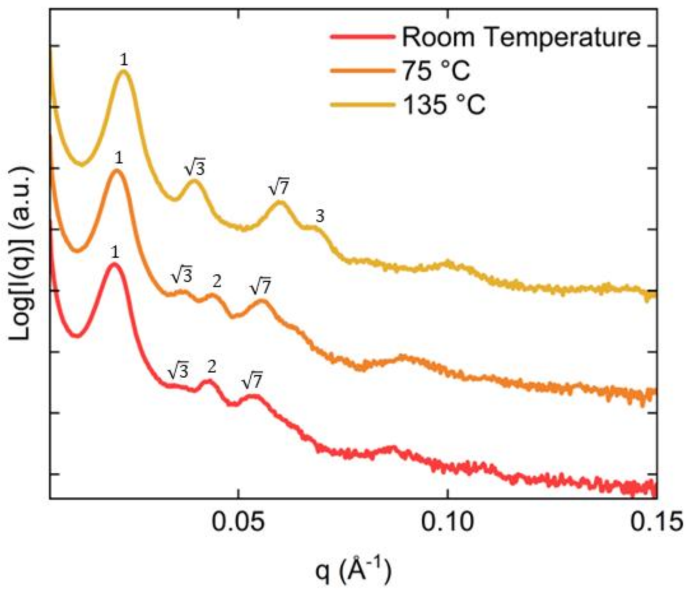

Figure S13. SAXS profiles of b-120 at room temperature, 75 °C, and 135 °C, all showing cylinders arranged in a hexagonal lattice.

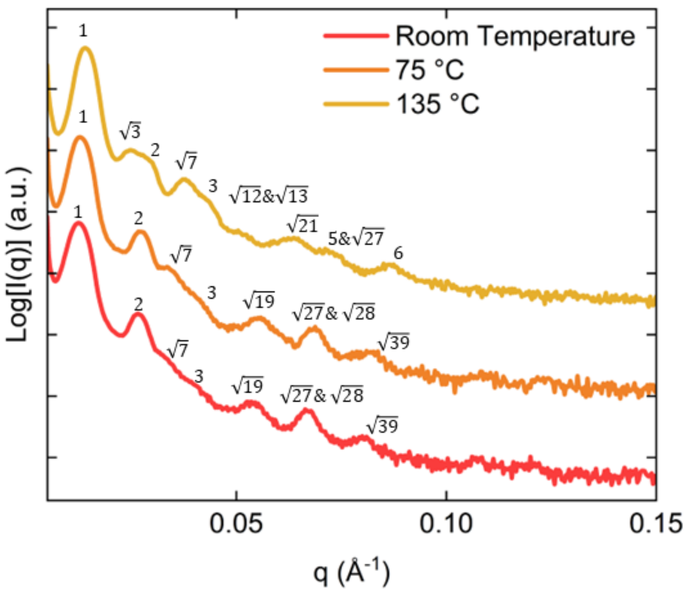

Figure S14. SAXS profiles of b-220 at room temperature, 75 °C, and 135 °C, all showing cylinders arranged in a hexagonal lattice.

Atomic Force Microscopy (AFM) nanomechanical testing

AFM measurements were conducted in a closed chamber with a controlled relative humidity of 29%, achieved by placing desiccants in the chamber overnight and maintaining them throughout the AFM experiments. All AFM samples were prepared via solvent casting using methanol as the solvent and stainless-steel plates as the substrate. The samples were first dried at ambient conditions for a few hours and then dried in a vacuum oven at 30 °C for 1~2 days to remove residual solvent.

To examine the temporal variation in the stiffness mapping of the cylindrical phase and the matrix upon air exposure, particularly over the duration of the tests presented in the manuscript, additional measurements were conducted on sample b-120. The results confirmed that, in a freshly prepared sample (within 10 min of exposure to air), the cylinders exhibited lower stiffness than the surrounding matrix. Stiffness mapping was repeated twice at the same location after 20 and 40 minutes of exposure under ambient conditions. As noted, the overall stiffness decreased with time; however, the morphology remained an inverse hexagonal structure (Figure S15), with the continuous phase remaining stiffer than the cylinders. Moreover, we hypothesize that the inverse HEX phase is critical in improving creep resistance and processability. If it were the standard HEX phase, the mechanical properties would be worse or similar to S-I or S-EB diblock copolymers.

Note that the slope fit in Figure S15 reflects the effective bulk stiffness, as the AFM probe deforms the polymer surface during indentation. The relationship between the slope value and the polymer's elastic modulus, as derived from the Hertz contact model, is given by:

$$\text{Slope fit} = 2\left(\frac{1-{\nu_{tip}}^2}{E_{tip}} + \frac{1-{\nu_{polymer}}^2}{E_{polymer}}\right)^{-1} \cdot \sqrt{R\delta} \qquad (S2)$$

where E_{tip} and $E_{polymer}$ represent the Young's moduli of the AFM probe and the polymer ($E_{tip} \gg E_{polymer}$), respectively, while ν_{tip} and $\nu_{polymer}$ are the Poisson's ratios for the AFM probe and the polymer. R represents the probe tip radius, and δ is the indentation depth.

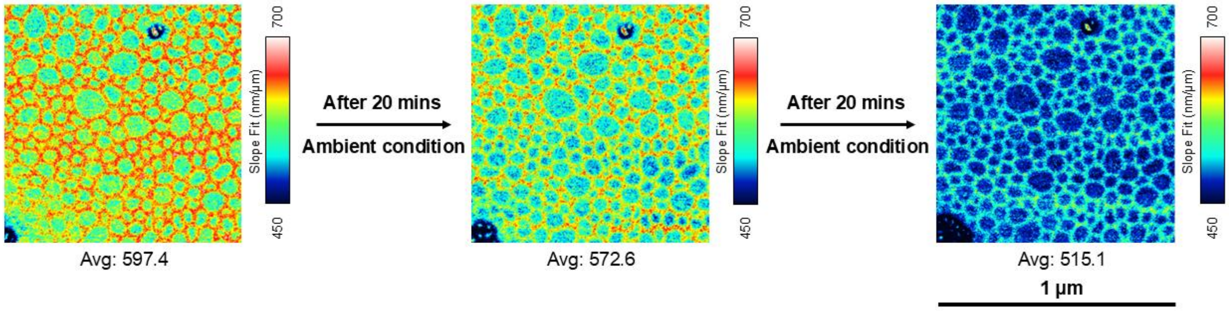

Figure S15. AFM slope fit mapping of the b-120 fresh sample (average slope with less than 10 mins exposure to air: 597.4 nm/µm), same sample after 20 minutes (average slope: 572.6 nm/µm) and 40 minutes (average slope: 515.5 nm/µm) exposure to air, with relative humidity of 29% in a closed chamber.

Calculation Details

Layer thickness of Glassy PATMAC domains

Assuming incompressibility, the volume fraction of the charged block in b-120 was calculated based on the density and the molecular weight of each block (DP of each block was obtained from ^{1}H NMR) (equation S3). The density of PiBA is close to 1.07 g/cm^3.[7] The density of PATMAC is estimated to be similar to the ATMAC monomer density, 1.17 g/cm^3 (estimated from the density of 80 wt% ATMAC in H_2O, which is 1.132 g/mL). Based on the calculation, we determined that the volume fraction of PATMAC block is 0.23~0.24 and that of PiBA block is 0.76~0.77. The calculation process is the same for r-100 and b-220.

$$Volume\ fraction = \frac{{}^{Mw_A}/_{\rho_A}}{{}^{Mw_A}/_{\rho_A} + {}^{Mw_B}/_{\rho_B}} \qquad (S3)$$

To obtain the diameter of PiBA cylinder located in the hexagonal lattice, we used cylinder-to-cylinder distance $L = d_{10}/\frac{\sqrt{3}}{2} = 35.3\ nm$ to calculate the area of a hexagon, which is 1,079.15 nm^2. We then used PiBA volume fraction 0.76 to calculate the base area of the PiBA cylinder, which is 820.15 nm^2. Knowing the area, we could calculate the diameter is 32.3 nm. With that, the glassy PATMAC layers separating adjacent cylinders can be as thin as 3 nm (35.3 nm – 32.3 nm) for b-120.

Flory Huggins Parameter and Microphase separation

To estimate the Flory-Huggins parameter (χ), we assume that the b-120 cylindrical phase Gibbs free energy is located at the order-disorder phase boundary, which would give us the lower bound of χ. The χ estimation was conducted under mean-field theory framework assuming the polymer is incompressible. The Gibbs free energy of the ordered phase can be expressed by equation S4

$$\frac{\Delta G_{ordered}}{kT} = \frac{\Sigma}{b^2}\left(\frac{\chi}{6}\right)^{\frac{1}{2}} + \frac{3}{8}\frac{d^2}{Nb^2} \qquad (S4)$$

where Σ is interfacial area per chain, *b* is segmental length, χ is Flory-Huggins parameter, and *d* is cylinder-to-cylinder distance. In the cylindrical structure, the number of chains per cell can be presented as $\frac{\frac{\sqrt{3}}{2}d^2L}{Nb^3}$, where L is the length of cylinders. Assuming the volume fraction of cylinder in hexagonal structure is *f*, we can obtain the radius of cylinder $R_c = 0.525f^{1/2}d$. The interfacial area per cell can be expressed as $\Sigma_{cell} = 2\pi R_c L$, and by dividing number of chains per cell, we

can obtain interfacial area per chain $\Sigma = 3.809\frac{f^{1/2}Nb^3}{d}$. With the information, Gibbs free energy of the ordered phase can be expressed as equation S5

$$\frac{\Delta G_{ordered}}{kT} = 1.555\frac{f^{\frac{1}{2}}Nb\chi^{\frac{1}{2}}}{d} + \frac{3}{8}\frac{d^2}{Nb^2} \qquad (S5)$$

By assuming that Gibbs free energy of the b-120 cylindrical phase lies at the order–disorder phase boundary, we obtain the Gibbs free energy at transition point as $\frac{\Delta G_{ordered}}{kT} = 1.83f^{\frac{1}{3}}(\chi N)^{\frac{1}{3}}$ and set $\Delta G_{ordered} = \Delta G_{disordered} = (\chi N)(1-f)f$. Thus, the relationship between volume fraction, χ and degree of polymerization (N) can be expressed in equation S6

$$(\chi N) = \left(\frac{1.83f^{\frac{1}{3}}}{(1-f)f}\right)^{\frac{3}{2}} \qquad (S6)$$

In b-120, the volume fraction of cylinder f is 0.76 (PiBA block is the cylindrical soft domain). Therefore, we get $(\chi N) = 27.7$. With $N = 117.6$, we can determine that $\chi = 0.236$. It should be noted that the actual χ may be much larger than 0.236, since the calculation is based on the assumption that Gibbs free energy of b-120 cylindrical phase lies at the order-disorder transition boundary. In fact, charge-neutral diblock copolymers are usually highly incompatible, and the χ between the two blocks can be as large as 10.[8]

Fracture healing process after hot press

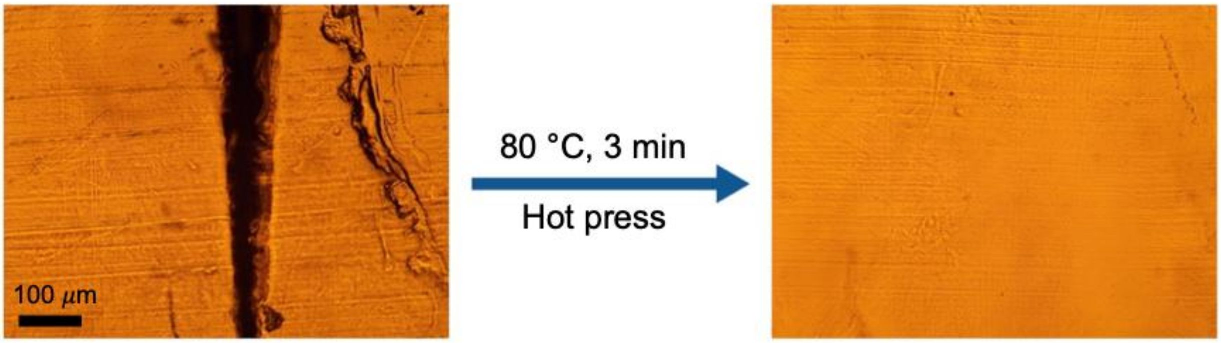

Figure S16. Fracture healing process after hot press monitored by an optical microscope.

References

(1) Tsai, C.-C.; Xu, J.; Duclos, C.; Xie, S. Microstructure and Viscoelasticity of Oppositely Charged Ionomer Blend Melts. *Macromolecules* **2025**, *58* (3), 1608–1620.

(2) Jullian, N.; Leonardi, F.; Grassl, B.; Peyrelasse, J.; Derail, C. Rheological Characterization and Molecular Modeling of Poly(n-Butyl Acrylate). *Applied Rheology* **2010**, *20* (3), 33685.

(3) Fredrickson, G. H.; Bates, F. S. Dynamics of Block Copolymers: Theory and Experiment. *Annual Review of Materials Research* **1996**, *26* (Volume 26,), 501–550.

(4) Ryu, C. Y.; Lee, M. S.; Hajduk, D. A.; Lodge, T. P. Structure and Viscoelasticity of Matched Asymmetric Diblock and Triblock Copolymers in the Cylinder and Sphere Microstructures. *Journal of Polymer Science Part B: Polymer Physics* **1997**, *35* (17), 2811–2823.

(5) Kossuth, M. B.; Morse, D. C.; Bates, F. S. Viscoelastic Behavior of Cubic Phases in Block Copolymer Melts. *Journal of Rheology* **1999**, *43* (1), 167–196.

(6) Rosedale, J. H.; Bates, F. S. Rheology of Ordered and Disordered Symmetric Poly(Ethylenepropylene)-Poly(Ethylethylene) Diblock Copolymers. *Macromolecules* **1990**, *23* (8), 2329–2338.

(7) Liu, S.; Wu, S.; Chen, Q. Using Coupling Motion of Connecting Ions in Designing Telechelic Ionomers. *ACS Macro Lett.* **2020**, *9* (7), 917–923.

(8) Bates, F. S.; Hillmyer, M. A.; Lodge, T. P.; Bates, C. M.; Delaney, K. T.; Fredrickson, G. H. Multiblock Polymers: Panacea or Pandora's Box? *Science* **2012**, *336* (6080), 434–440.
